# WNK1 kinase and its partners Akt, SGK1 and NBC-family Na^+^/HCO3^−^ cotransporters are potential therapeutic targets for glioblastoma stem-like cells linked to Bisacodyl signaling

**DOI:** 10.18632/oncotarget.25509

**Published:** 2018-06-05

**Authors:** Wanyin Chen, Leonel Nguekeu Zebaze, Jihu Dong, Laëtitia Chézeau, Perrine Inquimbert, Sylvain Hugel, Songlin Niu, Fréderic Bihel, Emmanuel Boutant, Eléonore Réal, Pascal Villa, Marie-Pierre Junier, Hervé Chneiweiss, Marcel Hibert, Jacques Haiech, Marie-Claude Kilhoffer, Maria Zeniou

**Affiliations:** ^1^ Laboratoire d’Innovation Thérapeutique, Centre National de la Recherche Scientifique/Université de Strasbourg, UMR7200, Laboratoire d’Excellence Medalis, Faculté de Pharmacie, Illkirch 67401, France; ^2^ Institut des Neurosciences Cellulaires et Intégratives, UPR3212, Centre National de la Recherche Scientifique, 67084 Strasbourg, France; Université de Strasbourg, Strasbourg 67084, France; ^3^ Laboratoire de Bioimagerie et Pathologies - LBP, UMR7021, Centre National de la Recherche Scientifique/Université de Strasbourg, Faculté de Pharmacie, Illkirch 67401, France; ^4^ Plateforme de Chimie Biologie Intégrative (PCBIS), Université de Strasbourg/CNRS UMS 3286, Laboratoire d’Excellence Medalis, ESBS Pôle API-Bld Sébastien Brant, Illkirch 67401, France; ^5^ Neuroscience Paris Seine-IBPS, CNRS UMR 8246/Inserm U1130/UPMC UMCR18, Paris 75005, France

**Keywords:** Bisacodyl/DDPM, glioblastoma cancer stem-like cells, WNK1, Akt/SGK1, NBC Na^+^/HCO3^−^ cotransporters

## Abstract

Glioblastoma is a highly heterogeneous brain tumor. The presence of cancer cells with stem-like and tumor initiation/propagation properties contributes to poor prognosis. Glioblastoma cancer stem-like cells (GSC) reside in hypoxic and acidic niches favoring cell quiescence and drug resistance. A high throughput screening recently identified the laxative Bisacodyl as a cytotoxic compound targeting quiescent GSC placed in acidic microenvironments. Bisacodyl activity requires its hydrolysis into DDPM, its pharmacologically active derivative. Bisacodyl was further shown to induce tumor shrinking and increase survival in *in vivo* glioblastoma models. Here we explored the cellular mechanism underlying Bisacodyl cytotoxic effects using quiescent GSC in an acidic microenvironment and GSC-derived 3D macro-spheres. These spheres mimic many aspects of glioblastoma tumors *in vivo*, including hypoxic/acidic areas containing quiescent cells. Phosphokinase protein arrays combined with pharmacological and genetic modulation of signaling pathways point to the WNK1 serine/threonine protein kinase as a mediator of Bisacodyl cytotoxic effect in both cell models. WNK1 partners including the Akt and SGK1 protein kinases and NBC-family Na^+^/HCO3^−^ cotransporters were shown to participate in the compound’s effect on GSC. Overall, our findings uncover novel potential therapeutic targets for combatting glioblastoma which is presently an incurable disease.

## INTRODUCTION

Glioblastoma (GBM) is the most common type of malignant brain tumor in adults with a reported incidence of 3–4 cases per 100,000 and a gender preference for male adults [1]. The median survival of GBM patients without treatment is of 3 months. The standard of care, consisting in surgery, radiotherapy and adjuvant temozolomide (TMZ) DNA alkylating chemotherapy, increases average survival only to 9–19 months. Less than 4.7% of patients survive 5 years after diagnosis [1, 2].

Tumor cell subpopulations with enhanced ability to initiate, propagate and maintain tumors have been identified in many cancers, including GBM [3, 4]. Based on their properties, such cancer cells have been designated as tumor initiating/propagating cells. Initially proposed to derive from malignant transformation of normal stem cells, these cells are also called cancer stem or stem-like cells. The hallmark of glioblastoma and other tumor stem-like cells (GSC) is their ability to recapitulate the heterogeneity and complexity of tumors from which they are derived following serial orthotopic transplantation *in vivo* [5–7]. This GSC property is supported by their long-term, self-renewal ability, their capacity to divide symmetrically or asymmetrically, as well as their ability to differentiate into low tumorigenic cancer cells [8]. Interestingly, slow-growing quiescent cell populations with *in vivo* tumorigenic potential have been reported within human GBM [9], and a single-cell RNA-seq analysis has identified co-expression of stemness and quiescent-cell molecular markers in cells directly sampled from patients’ glioblastoma [10].

Although initially believed to be a static cell subpopulation within tumors with invariable properties, cancer stem-like cells are now rather considered to correspond to a transient state that any tumor cell may acquire. Genetic and epigenetic determinants, as well as signaling cues emanating from the tumor microenvironment or therapeutic intervention have been proposed to drive acquisition or loss of cancer stem-like cell properties [11–15]. Several studies have pointed to hypoxic/acidic microenvironments as the ones of the niche of GSC. GBM contain hypoxic regions where quiescent glioblastoma cells have been localized [16]. Low oxygen conditions as well as acidic conditions were shown to facilitate GSC growth, survival, stemness and tumorigenic potential [17, 18]. The quiescent state, which may be reversed in the presence of appropriate environmental cues, is believed to be one of the major determinants of treatment resistance and tumor recurrence. For example, in glioblastoma animal models treated with TMZ, the quiescent GSC subpopulation survives and drives tumor regrowth through the production of rapidly dividing cells. Interestingly, ablation of these cells hinders tumor development [3]. Thus, novel therapeutic approaches targeting GSC-like cells in their quiescent state, within the tumor microenvironmental conditions (low oxygen and low pH), are promising approaches for GBM treatment.

Using *in vitro* experimental models of TMZ-resistant proliferating and quiescent GSC derived from GBM patients, we recently identified DDPM (4,4’-dihydroxydiphenyl-2-pyridyl-methane), as a cytotoxic compound inducing necrosis of GSC in a quiescent state whereas sparing proliferating GSC [19, 20]. DDPM is a hydrolysis derivative of the commonly used laxative drug Bisacodyl (4,4’-diacetoxydiphenyl-2-pyridyl-methane), and is responsible for all pharmacological actions of this compound. We further showed that microenvironment acidification of proliferating GSC induced cell quiescence and sensitized them to DDPM. Coherently, DDPM also kills quiescent cells located in the inner-layer of giant tumorospheres clonally derived from a single GSC. These 3D clonal macro-spheres, also called “organoids” [21], recapitulate many histological aspects of GBM tumors *in vivo*, including development of necrotic areas. Finally, an *in vivo* antitumoral activity of Bisacodyl was demonstrated in orthotopic xenograft mouse models of GBM [19].

In this report, we demonstrate that DDPM exerts its cytotoxic effects by altering the mobilization of the serine/threonine protein kinase WNK1 (With no- lysine (K) kinase 1). WNK1 is one of the four members of the WNK protein family. WNK1 functions depend on its phosphokinase activity and/or scaffolding with protein partners [22]. They have been associated to a variety of cellular processes, including fluid and electrolyte homeostasis, cell proliferation, migration and survival, as well as vesicular trafficking and autophagy. Mutations in the *WNK1* and *WNK4* genes have been associated with inherited forms of hypertension [23–25]. WNK1 expression has been reported in patients’ glioblastoma and shown to modulate the activity of ion cotransporters of the NKCC family in primary glioblastoma cell lines leading to improved cell volume regulation and enhanced cell resistance to TMZ and cell motility [26].

Our data show that DDPM inhibits the activity of a kinase cascade constituted by WNK1 and its upstream regulators AKT and SGK1 (Serum and glucocorticoid-stimulated protein kinase-1). This results in subsequent stimulation of the activity of NBC Na^+^/HCO3^−^ cotransporters which are known targets of WNK1. Our results uncover novel, potentially interesting therapeutic targets for the treatment of GBM which is to date an incurable disease.

## RESULTS

### DDPM modifies the phosphorylation status of WNK1 T60 in quiescent GSC in an acidic environment

**Figure 1:**
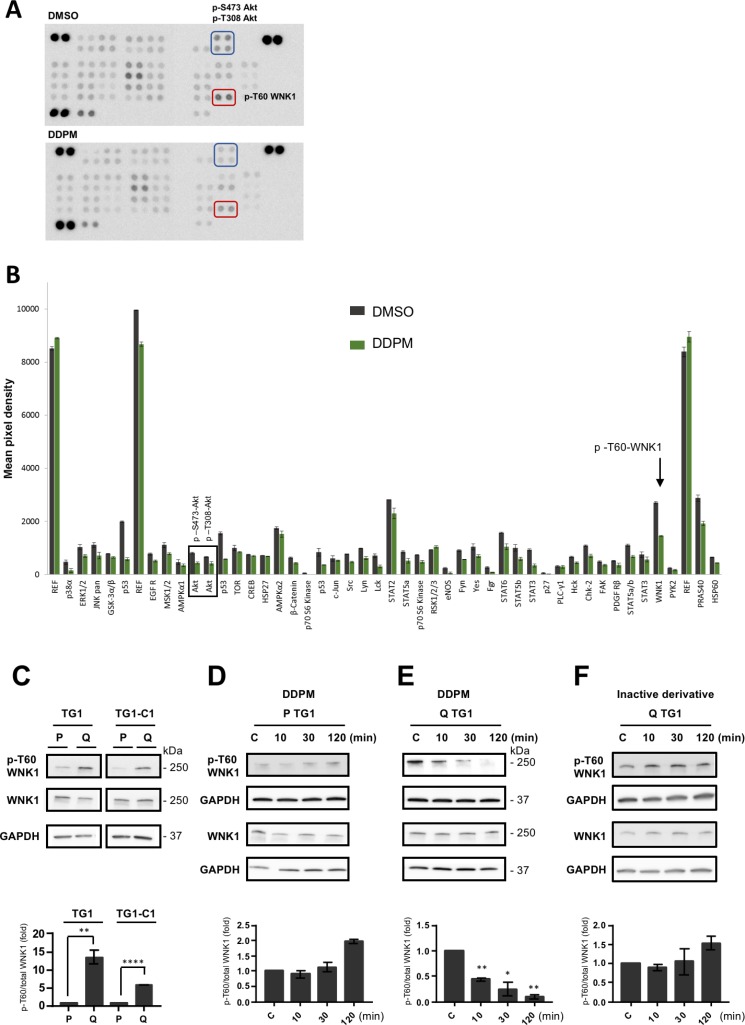
DDPM modifies the phosphorylation status of WNK1 T60 in quiescent GSC in an acidic environment (**A**) Phospho-kinase Array membranes from R&D Systems were incubated with protein extracts from quiescent TG1-C1 GSC treated with 10 µM of DDPM (in 1% DMSO, lower panel) or DMSO alone (vehicle, upper panel) for 2 hours. The phosphorylation of WNK1-T60 was significantly decreased by DDPM (compare spots in red rectangles in upper and lower panels). Akt S473 and T308 phosphorylation levels were also decreased by DDPM (compare spots in blue rectangles in upper and lower panels). (**B**) Histogram representing phospho-kinase array results from TG1-C1 GSC treated with 10 µM of DDPM (in 1% DMSO; green bars) or DMSO alone (grey bars). Results are shown as the mean pixel intensity of the signal from triplicates within the same experiment (± s.d.), analyzed with the ImageJ software. The phosphorylation of WNK1-T60 was significantly decreased by DDPM (black arrow). Black rectangle illustrates modifications in the phosphorylation status of Akt S473 and T308 required for Akt activation. Activated Akt was shown to phosphorylate T60 in WNK1. (**C**) WNK1 and phospho (p)-T60 WNK1 levels were determined by Western blotting and appropriate antibodies in extracts from proliferating (P) and quiescent (Q) TG1 and TG1-C1 GSC. GAPDH expression was used as a loading control. Blots are representative out of at least two independent experiments. Quantification plot representing p-T60 WNK1 levels relative to total WNK1 protein is shown (± SEM; *n* ≥ 2). Relative p-T60/total WNK1 levels in quiescent TG1 and TG1-C1 cells were normalized to levels observed in proliferating TG1 and TG1-C1 GSC, respectively. Student *t*-test. ^**^p < 0.005, ^****^p < 0.0001. (**D**–**F**) Proliferating (P) and quiescent (Q) TG1 GSC were treated with DMSO (1%, C: control) or with DDPM (10 µM in 1% DMSO) for 10, 30 or 120 minutes (D and E, respectively). Quiescent (Q) TG1 GSC were treated with 1% DMSO (C) or with an inactive derivative of DDPM (10 µM in 1% DMSO) for 10, 30 or 120 minutes (F). Protein extracts from these cells were subjected to Western blotting to determine relative p-T60/total WNK1 levels. GAPDH was the loading control. Blots are representative out of at least three independent experiments. Quantification plots under each blot are as in C (± SEM; *n* ≥ 2). Statistical analysis was performed by pairwise comparison of results at each time point relatively to control (C) conditions used for normalization. Student *t*-test. ^*^p < 0.05, ^**^p < 0.005.

All experiments were performed on TG1 and TG1-C1 GSC sub-clones isolated from GBM patient biopsies. The Bisacodyl’s active derivative DDPM is cytotoxic for quiescent GSC present in slightly acidic culture conditions (pH ∼6.6) whereas it spares proliferating cells maintained in physiological pH conditions (pH ∼7.4) [19, 20]. We first determined whether DDPM affects the phosphorylation levels of several kinases and kinase substrates in quiescent GSC with an antibody array directed against 43 distinct kinase phosphorylation sites (human phospho-kinase array kit membranes R&D system). A 2-hour DDPM (10 µM) treatment of quiescent GSC altered phosphorylation of several kinases and kinase substrates compared to DMSO-treated cells (Figure 1A and 1B). In coherence with DDPM cytotoxic effects, we observed a decrease in the phosphorylation levels of ERK1/2, Akt, ß-catenin and STAT proteins previously shown to be involved in glioblastoma cell proliferation, survival and/or stem-like properties. Interestingly, we observed a (∼40%) decrease in the phosphorylation levels of the WNK1 kinase (p-T60-WNK1) (Figure 1A and 1B), previously unsuspected to be involved in the control of GSC properties. Western blot analyses showed that although WNK1 was expressed in both quiescent and proliferating GSC (Figure 1C), its phosphorylated form was enriched in quiescent GSC (Figure 1C). Furthermore, the ratio of p-T60 WNK1 against total WNK1 did not change in proliferating GSC following DPPM treatment (Figure 1D and Supplementary Figure 1A, left panel). On the contrary, DPPM strongly inhibited in a time-dependent manner, WNK1 phosphorylation in quiescent GSC, p-T60 WNK1 becoming undetectable after 120 min of treatment (Figure 1E and Supplementary Figure 1A, middle panel). Conversely, an inactive derivative of DDPM, LPI3271 (Supplementary Figure 2) was without effect on WNK1 phosphorylation (Figure 1F and Supplementary Figure 1A, right panel). Altogether these results suggest a DDPM activity-dependent reduction of p-T60 WNK1 levels in quiescent GSC maintained in an acidic environment.

### DDPM modifies the catalytic activity of WNK1 both in proliferating and quiescent GSC

**Figure 2:**
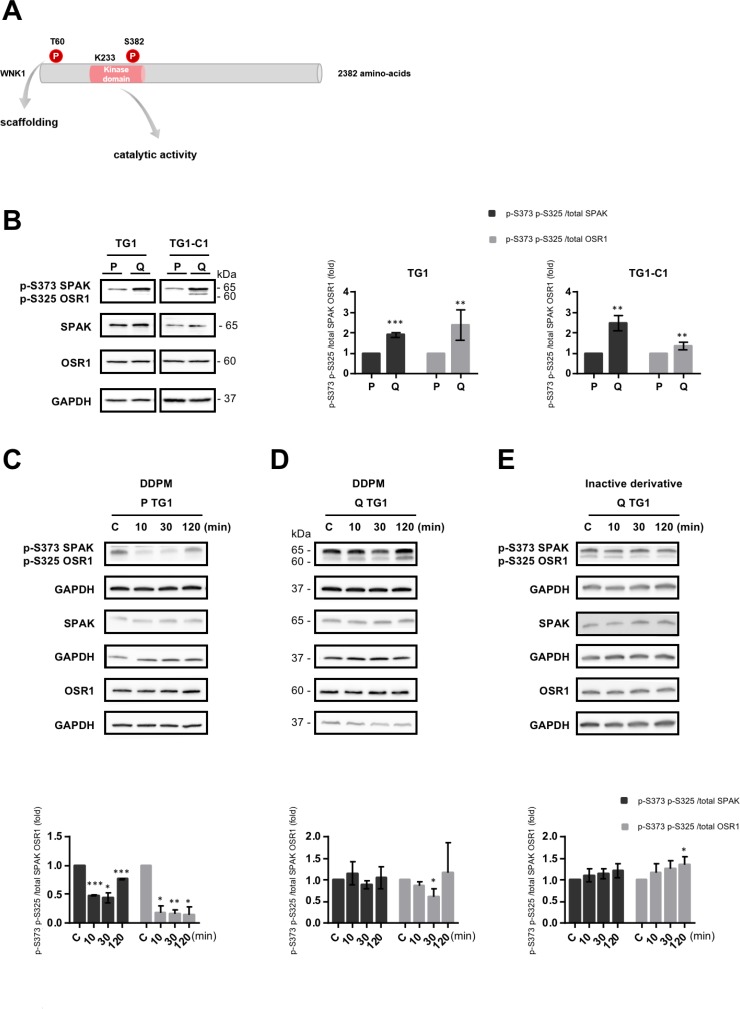
DDPM modifies the catalytic activity of WNK1 both in proliferating and quiescent GSC (**A**) Schematic representation of human WNK1. Threonine 60 (T60) whose phosphorylation modulates WNK1 functions depending on scaffolding with protein partners is shown. The catalytic activity of the enzyme is dependent on the phosphorylation status of serine 382 (S382) within the kinase domain and requires a lysine at position 233 (K233) involved in ATP-binding. (**B**) SPAK and OSR1 as well as p-S373 SPAK and p-S325 OSR1 protein levels were analyzed by Western blotting in proliferating (P) and quiescent (Q) TG1 and TG1-C1 GSC with GAPDH as loading control. Quantification plots represent p-SPAK/OSR1 protein levels relative to total SPAK (dark gray bars) or to total OSR1 (light grey bars) in quiescent (Q) TG1 and TG1-C1 normalized to proliferating (P) cells (± SEM; *n* ≥ 2). Student *t*-test. ^**^p < 0.005, ^***^p < 0.0005. (**C**–**E**) Western blot results with anti-p-SPAK/OSR1 or anti-SPAK or OSR1 antibodies in proliferating (P) and quiescent (Q) TG1 GSC treated with DMSO (1%, C: control) or with DDPM (10 µM in 1% DMSO) for 10, 30, or 120 minutes (C and D, respectively), or in quiescent (Q) TG1 GSC treated with DMSO (1%, C: control) or an inactive derivative of the compound (10 µM in 1% DMSO) for 10, 30 or 120 minutes (E). Quantification plots are as in B (± SEM; *n* ≥ 3). Statistical analysis was performed by pairwise comparison of results at each time point relatively to control (C) conditions used for normalization. Student *t*-test. ^*^p < 0.05, ^**^p < 0.005; ^***^p < 0.0005.

In addition to its functions involving scaffolding with protein partners which may be mediated by T60 phosphorylation (Figure 2A) [27], WNK1 operates in cells as a kinase. WNK1 catalytic activation requires auto-phosphorylation of at least one serine residue (S382 in human WNK1) within the activation loop (Figure 2A). The catalytic lysine K233 (Figure 2A) is also required for phosphotransferase activity. WNK1 activities (scaffolding and catalytic activation) may be associated or function independently [22].

To determine whether the catalytic function of WNK1 is also affected by DDPM, the kinase activity of the enzyme was measured indirectly by determining the phosphorylation status of two WNK1 substrates in other cell systems, *i.e.* SPAK (STE20/SPS1-related proline/alanine-rich kinase) and OSR1 (Oxidative stress-responsive kinase 1) [28]. Using a WNK1 shRNA-based targeting approach as well as the WNK kinase inhibitor WNK463 [29], we first verified that SPAK and OSR1 proteins (respectively S373 in SPAK and S325 in OSR1) were also WNK substrates in TG1 and TG1-C1 GSC (Supplementary Figure 3A and 3B). As presented in Figure 2B, we further show that higher p-S373 SPAK/p-S325 OSR1 levels normalized respectively to total SPAK (dark grey bars) or to OSR1 (light gray bars) are detected in quiescent TG1 and TG1-C1 GSC compared to proliferating cells. DDPM treatment resulted in a transient decrease in the relative p-SPAK/OSR1 levels in proliferating TG1 and TG1-C1 cells (Figure 2C and Supplementary Figure 1B left panel). A weak, transient, yet statistically significant decrease in phosphorylated SPAK/OSR1 normalized to total OSR1 protein was also observed in quiescent TG1 and TG1-C1 cells after a 30-min treatment with DDPM (10 µM) (Figure 2D and Supplementary Figure 1B, middle panel). Furthermore, a similar decrease was not detected in quiescent TG1 and TG1-C1 treated with the inactive DDPM derivative (Figure 2E and Supplementary Figure 1B right panel).

Altogether these results suggest that the basal catalytic activity of WNK1 is higher in quiescent GSC compared to proliferating GSC. DDPM treatment results in a transient decrease in SPAK/OSR1 phosphorylation, thus implying lower WNK1 catalytic activity both in conditions in which the compound is active (quiescent GSC in slightly acidic environment) and in conditions in which the molecule is not cytotoxic (proliferating GSC at physiological pH). However, DDPM effects on WNK1 catalytic activity were accentuated in proliferating GSC compared to quiescent GSC.

### DDPM cytotoxicity on GSC-derived macro-spheres depends on WNK1 levels, T60 phosphorylation and catalytic activity

To determine the impact of WNK1 modulation on DDPM cytotoxicity, we first performed transient expression of the following FLAG-tagged wild-type and mutant WNK1 constructs in proliferating and quiescent TG1 and TG1-C1 GSC: empty vector, wild-type WNK1 (WT), T60 non-phosphorylable WNK1 (T60A), T60 phosphomimetic mutant WNK1 (T60D/E) and catalytically inactive WNK1 (K233M) (Figure 2A). Cellular localization of the expressed proteins was examined by FLAG immunofluorescent staining and confocal microscopy imaging. WNK1 mutations did not alter subcellular distribution of the protein. The same punctate staining evenly distributed in the cytoplasm was observed in all conditions even though the expression levels of WT and mutant WNK1 proteins were generally lower in quiescent compared to proliferating GSC. Punctate WNK1 staining was also present in the nucleus of some cells (Supplementary Figure 4 and data not shown). This staining pattern is similar to the previously reported distribution of overexpressed or endogenous WNK1 in other cell systems [30, 31].

**Figure 3:**
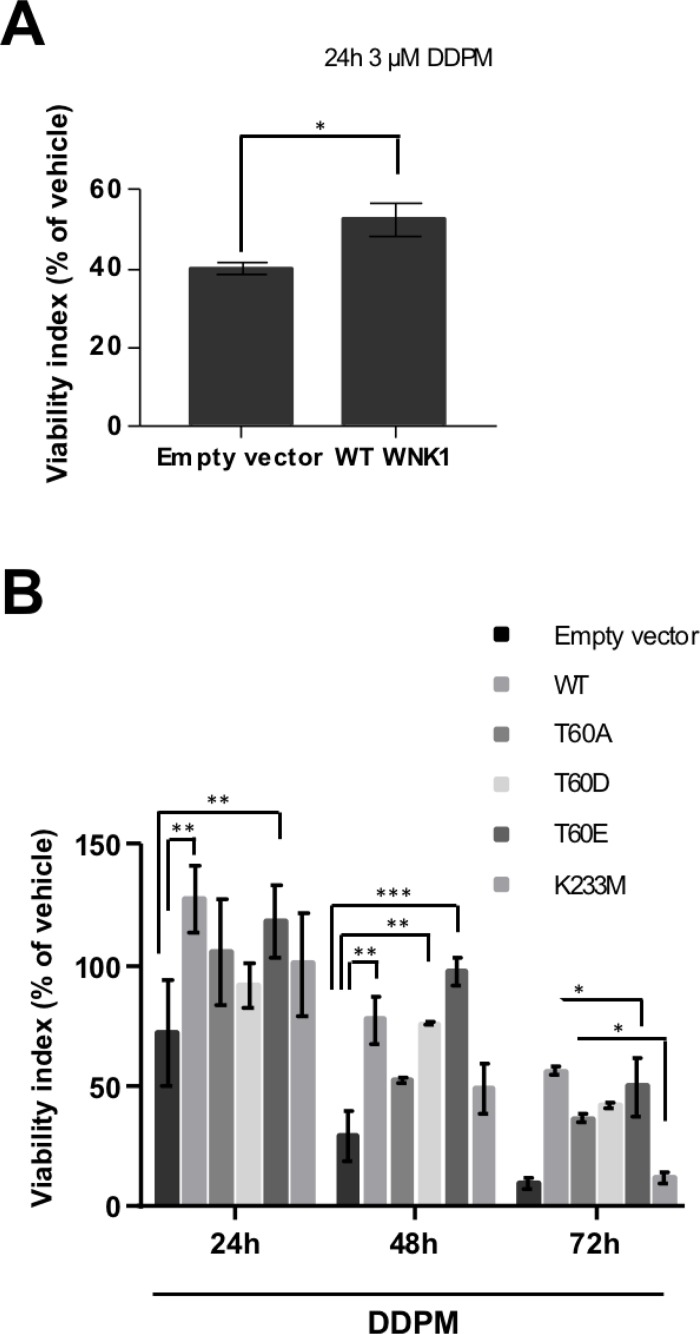
DDPM cytotoxicity on GSC-derived macro-spheres depends on WNK1 levels, T60 phosphorylation and catalytic activity (**A**) 3D macro-spheres were obtained from control TG1 GSC (Empty vector) or TG1 GSC stably overexpressing wild-type (WT) WNK1. 8 individual spheres of each type (and of similar size) were treated with 1% DMSO or DDPM (3 µM in 1% DMSO) for 24 hours. Cell viability was evaluated following treatment with the 3D ATP-Glo assay and expressed (as a percentage) for each type of sphere with respect to DMSO alone-treated controls. Error bars represent the mean (± SEM) (*n =* 8 spheres for each condition). Student *t*-test. ^*^p < 0,05. Mann Whitney non-parametric two-tailed test: *p* = 0.007. (**B**) Neurospheres derived from control (Empty vector) TG1 GSC or TG1 GSC stably overexpressing WT or mutant (T60A, T60D, T60E, K233M) forms of WNK1 were treated with DMSO alone (1%) (control) or 1% DMSO in the presence of 2 µM of DDPM for 24, 48 and 72 hours. Cell viability was evaluated following treatment with the 3D ATP-Glo assay and expressed (as a percentage) for each type of sphere with respect to DMSO alone-treated corresponding controls. Error bars represent the mean (± SEM) (*n =* 3 spheres for each condition and each time point). Two-way ANOVA and Tukey’s multiple comparisons test. ^*^p < 0.05, ^**^p < 0.005, ^***^p < 0.0005.

TG1-derived cell lines stably transfected with empty vector, WT or mutant WNK1 constructs were then established (Supplementary Figure 5A). Overexpression of WT or mutant WNK1 proteins did not alter EdU incorpororation properties in these cells (Supplementary Figure 5B) which were subsequently used to examine the impact of WNK1 modulation on DDPM cytotoxicity on GSC. Macro-spheres derived from these cells, previously developed and characterized in our laboratory and shown to contain large numbers of quiescent GSC [19], were treated with 3 µM of DDPM or with vehicle (1% DMSO) for 24 hours. Images of representative macro-spheres at the end of the 24-hour treatment with DMSO or with DDPM are shown in Supplementary Figure 6. Cell viability was then evaluated by measuring ATP levels. As expected, DDPM decreased cell viability in macro-spheres derived from control GSC (Figure 3A). Overexpression of WT WNK1 resulted in a decreased cytotoxic effect of DDPM compared to control cell-derived macro-spheres (Figure 3A). Finally, DDPM activity was evaluated at 24, 48 and 72 hours of treatment of macro-spheres derived from TG1 cells stably overexpressing WT and mutant (T60A, T60D/E and K233M) forms of WNK1 and compared to the activity of the compound at the same time-points in empty vector-stably transfected cells assembled in macro-spheres (Figure 3B). 3 macro-spheres of each cell type were used for each condition (2 µM DDPM in 1% DMSO or 1% DMSO alone). Overexpression of the WT and T60 phosphomimetic mutants (T60D/E) significantly protected cells from DDPM activity compared to empty vector-transfected control cells. The protective effect was observed at all time-points for WT and T60E forms of WNK1 and was more pronounced for the T60E compared to the T60D mutant (Figure 3B). Altogether these data suggest that decreased T60 phosphorylation level is required for DDPM to affect GSC because an increase in T60-phosphorylated WNK1 protected these cells from the compound. Finally, no protection was seen when the K233M mutant was overexpressed in TG1 cells arguing that dynamic modifications such as a transient reduction in WNK1 catalytic activity observed in quiescent GSC in the presence of the compound (Figure 2D) may also mediate DDPM cytotoxicity in quiescent GSC.

### DDPM modifies the phosphorylation status of the WNK1 upstream regulators Akt and SGK1 in quiescent GSC

**Figure 4:**
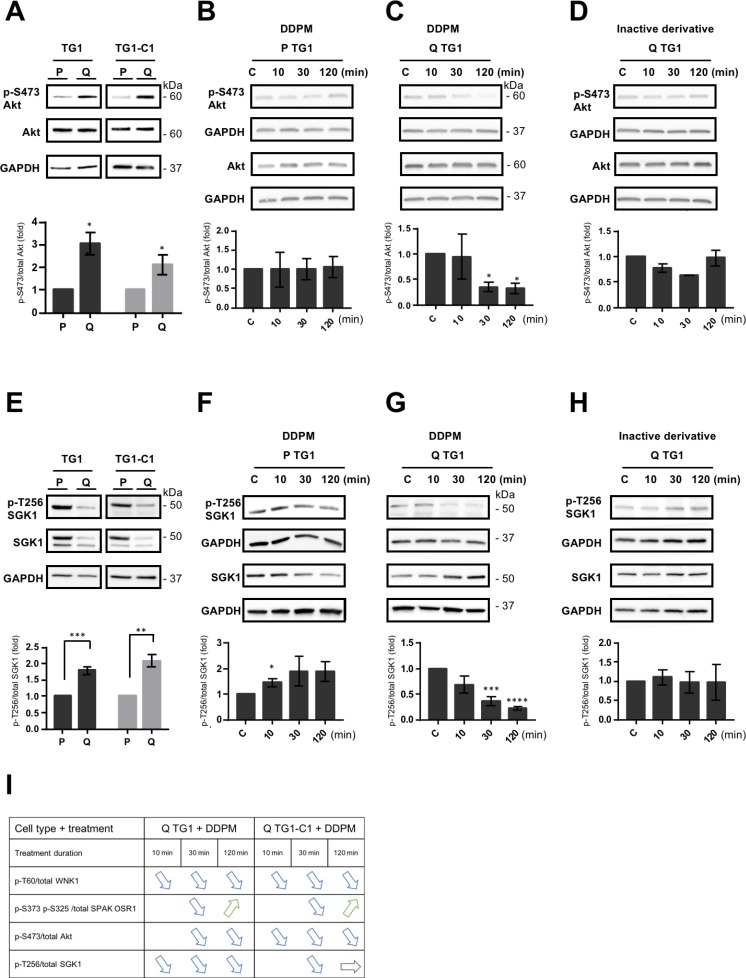
DDPM modifies the phosphorylation status of the WNK1 upstream regulators Akt and SGK1 in quiescent GSC (**A**) Relative p-S473/total Akt protein levels in proliferating (P) and quiescent (Q) TG1 and TG1-C1 GSC were analyzed by Western blotting, quantified with the ImageJ software and represented as histograms. The loading control was GAPDH. Results are representative of at least three independent experiments. Statistical analysis was performed by comparing expression levels in quiescent (Q) cells against proliferating (P) TG1 or TG1-C1 GSC. Student *t*-test. ^*^p < 0.05. (**B**–**D**) Western blotting was used to determine relative p-S473/total Akt levels in proliferating (P) and quiescent (Q) TG1 GSC treated with DMSO (1%, C: control) or with DDPM (10 µM in 1% DMSO) for 10, 30 or 120 minutes (B and C, respectively) or in quiescent (Q) TG1 GSC treated with 1% DMSO (C) or with an inactive derivative of DDPM (10 µM in 1% DMSO) for 10, 30 or 120 minutes (D). The loading control was GAPDH level and results are representative of at least two independent experiments. Quantification plots are presented as in A (± SEM; *n* ≥ 2). Statistical analysis was performed by pairwise comparison of results at each time point relatively to control (C) conditions used for normalization. Student *t*-test. ^*^p < 0.05. (**E**) Relative p-T256/total SGK1 protein levels in proliferating (P) and quiescent (Q) TG1 and TG1-C1 GSC were analyzed and represented as in A (± SEM; *n* ≥ 3). Student *t*-test. ^**^p < 0.005, ^***^p < 0.0005. (**F**–**H**) p-T256/total SGK1 protein levels were determined by Western blotting with appropriate antibodies in TG1 GSC treated in conditions as those in B, C and D, respectively. Quantification plots are represented as above (± SEM; *n* ≥ 2). Student *t*-test. ^*^p < 0.05, ^***^p < 0.0005, ^****^p < 0.0001. (**I**) Table resuming DDPM timing of action and effects on the phosphorylation status of WNK1 and WNK1 related proteins on specific residues in quiescent (Q) TG1 and TG1-C1 GSC.

The Akt and SGK1 kinases have been previously reported to phosphorylate WNK1 on T60, a step involved in kinase activity independent functions of WNK1 [27, 32, 33]. As indicated previously, reduced p-S473 and p-T308 Akt signals were observed in DDPM-treated quiescent GSC using phosphokinase arrays (Figure 1A and B). Using Western blot assays, we detected, as for WNK1, higher p-S473 Akt levels in quiescent TG1 and TG1-C1 GSC compared to proliferating cells (Figure 4A). DDPM induced a time-dependent decrease in p-S473 levels in quiescent TG1 and TG1-C1 cells (Figure 4C and Supplementary Figure 1C). In contrast, DDPM did not alter p-S473 Akt levels in proliferating GSC (Figure 4B and Supplementary Figure 1C). LPI3271, the inactive derivative of DDPM, did not significantly affect Akt phosphorylation in all conditions tested (Figure 4D and Supplementary Figure 1C).

Despite the presence of overall higher SGK1 expression in proliferating compared to quiescent GSC, the ratios of the phosphorylated active form of SGK1 (p-T256 SGK1) over total SGK1 were consistently higher in quiescent conditions (Figure 4E). DDPM induced a slight increase in p-T256 SGK1 levels in proliferating TG1 cells (Figure 4F). In contrast, DDPM inhibited p-T256 SGK1 relative levels in a time-dependent manner in quiescent TG1 cells (Figure 4G). A slight decrease in relative p-T256 SGK1 levels was also observed in quiescent TG1-C1 GSC (Supplementary Figure 1D left and middle panels). No variation in p-T256 SGK1 levels was detected in quiescent TG1 and TG1-C1 GSC treated with the inactive derivative of DDPM (Figure 4H and Supplementary Figure 1D, right panel).

These data show that DDPM inhibits the phosphorylation of WNK1, and of its upstream regulator Akt specifically in quiescent TG1 and TG1-C1 GSC, whereas its effect on SGK1 phosphorylation is more pronounced in the TG1 cell type. A table resuming levels and timing at which DDPM is acting in quiescent TG1 and TG1-C1 cells is presented in Figure 4I.

### Modulation of Akt and SGK1 impacts WNK1 T60 phosphorylation and DDPM cytotoxicity on GSC

**Figure 5:**
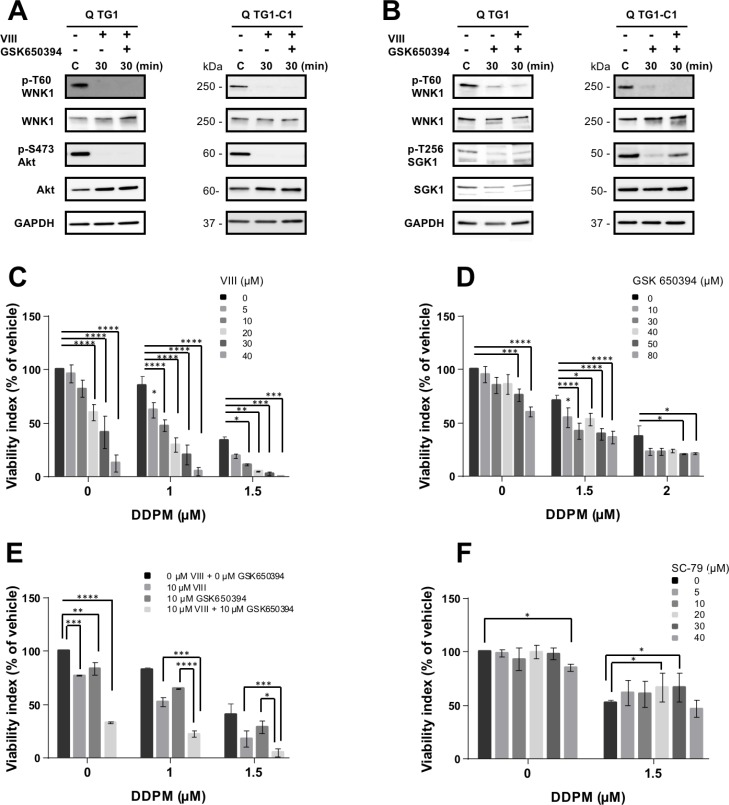
Modulation of Akt and SGK1 impacts WNK1 T60 phosphorylation and DDPM cytotoxicity on GSC (**A**, **B**) p-T60 WNK1 and WNK1, p-S473 Akt and Akt and p-T256 SGK1 and SGK1 protein levels were measured by Western blotting in quiescent (Q) TG1 and TG1-C1 GSC treated with vehicle (0.5% DMSO) or with Akt and/or SGK1 inhibitors VIII (TG1:10 µM, TG1-C1: 20 µM) and GSK 650394 (TG1: 30 µM and TG1-C1: 80 µM) for indicated times. GAPDH was the loading control. Results for TG1 GSC treated with the SGK1 inhibitor were obtained from samples loaded on nonconsecutive lanes of the same gel. (**C**–**E**) Quiescent TG1 GSC were either mock-treated (1% DMSO) or incubated in the presence of DDPM at indicated concentrations and/or Akt inhibitor VIII (C), SGK1 inhibitor GSK 650394 (D) or a combination of both compounds (E) for 24 hours. Cell viability measurements were performed with the ATP-based CellTiter-Glo^®^ cell viability assay. Results are expressed as a percentage of the viability of control TG1 GSC incubated with 1% DMSO alone (vehicle) for 24 hours. Results are the mean of three (C, D) or two (E) independent experiments performed in triplicate (± SEM). Statistical analysis was performed with two-way ANOVA and Tukey’s multiple comparisons test. ^*^p < 0.05, ^**^p < 0.005, ^***^p < 0.0005; ^****^p < 0.0001. (**F**) Quiescent TG1 GSC were incubated in 1% DMSO (controls) or pretreated for 24 hours with the indicated concentrations of the Akt activator SC-79. Indicated concentrations of DDPM (in 1% DMSO) were then added and treatment was pursued for 24 hours. Control cells were maintained in 1% DMSO. Cell viability assays, presentation of results and statistical analysis were performed as described in C, D and E. ^*^p < 0.05.

To determine whether Akt and/or SGK1 could be involved in WNK1 T60 phosphorylation in quiescent GSC, relative p-T60 WNK1 levels were evaluated in quiescent TG1 and TG1-C1 cells in which Akt and/or SGK1 function was inhibited by treating the cells with compounds VIII and/or GSK 650394, respectively. As shown in Figure 5A and 5B, both Akt and SGK1 inhibitors were able to reduce p-T60 WNK1 levels suggesting that these kinases may be responsible for WNK1 T60 phosphorylation status in quiescent GSC.

DDPM activity was then assayed in the presence of increasing concentrations of the Akt and SGK1 inhibitors or with combinations of both compounds, in quiescent TG1 and TG1-C1 GSC (Figure 5C–5E, respectively and Supplementary Figure 7A–7C). WNK1 inhibitor WNK463 was not used in this experiment since it is a pan-WNK kinase inhibitor targeting the catalytic activity of WNK1 and its effects on T60 phosphorylation and associated functions are unknown [29]. As shown in Figure 5C and 5D, a combined treatment of DDPM with either inhibitor significantly increased DDPM cytotoxicity in quiescent TG1 GSC even at inhibitor concentrations that alone are not cytotoxic. Simultaneous addition of both inhibitors reinforced DDPM cytotoxicity on quiescent TG1 GSC compared to DDPM with either inhibitor alone (Figure 5E). Akt inhibition also enhanced DDPM cytotoxicity in quiescent TG1-C1 at concentrations which alone do not affect cell viability (Supplementary Figure 7A), whereas only the highest concentration of the SGK1 inhibitor tested (80 µM) enhanced cytotoxicity (Supplementary Figure 7B). Moreover, 30 µM of the SGK1 inhibitor did not potentiate the enhancing effect of the Akt inhibitor on DDPM action in quiescent TG1-C1 GSC (Supplementary Figure 7C). Finally, in contrast to Akt inhibition, a pretreatment of TG1 and TG1-C1 GSC with the Akt activator SC-79 partially protected quiescent cells from DDPM cytotoxicity (Figure 5F and Supplementary Figure 7D). However, as observed for the other modulators of the Akt-SGK1-WNK1 signaling pathway, the protective effect of the Akt activator was again weaker on GSC from TG1-C1 compared to TG1 as illustrated in Figure 5C–5F and in Supplementary Figure 7.

Altogether these data establish a link between proteins of the Akt/SGK1/WNK1 signaling module and DDPM cytotoxicity both in quiescent TG1 and TG1-C1 GSC isolated from glioblastoma patients. Although there are some differences in the amplitudes of GSC responses to inhibitors between TG1 and TG1-C1 cells, this may be related to inherent characteristics of these cell types.

### DDPM induces quiescent GSC cell death by deregulating Na^+^/HCO3^−^ cotransporter cell surface expression and activity

Previous studies have shown that WNK1 may limit the surface expression and activity of NBCe1, an electrogenic Na^+^/HCO3^−^ cotransporter involved in ion and pH regulation by recruiting SPAK to the cotransporter [34]. This requires the N-terminal domain of WNK1 (WNK1^1–119^), but not its catalytic activation [35]. The electroneutral Na^+^/HCO3^−^ cotransporter NBCn1, that has been associated with cancer, could also be a downstream target of the WNK1/SPAK pathway [36]. We hypothesized that, DDPM reduction of WNK1 T60 phosphorylation in quiescent GSC might lead to an abnormal increase in NBCe1 and/or NBCn1 plasma membrane expression/activity, thereby causing cytotoxicity.

**Figure 6:**
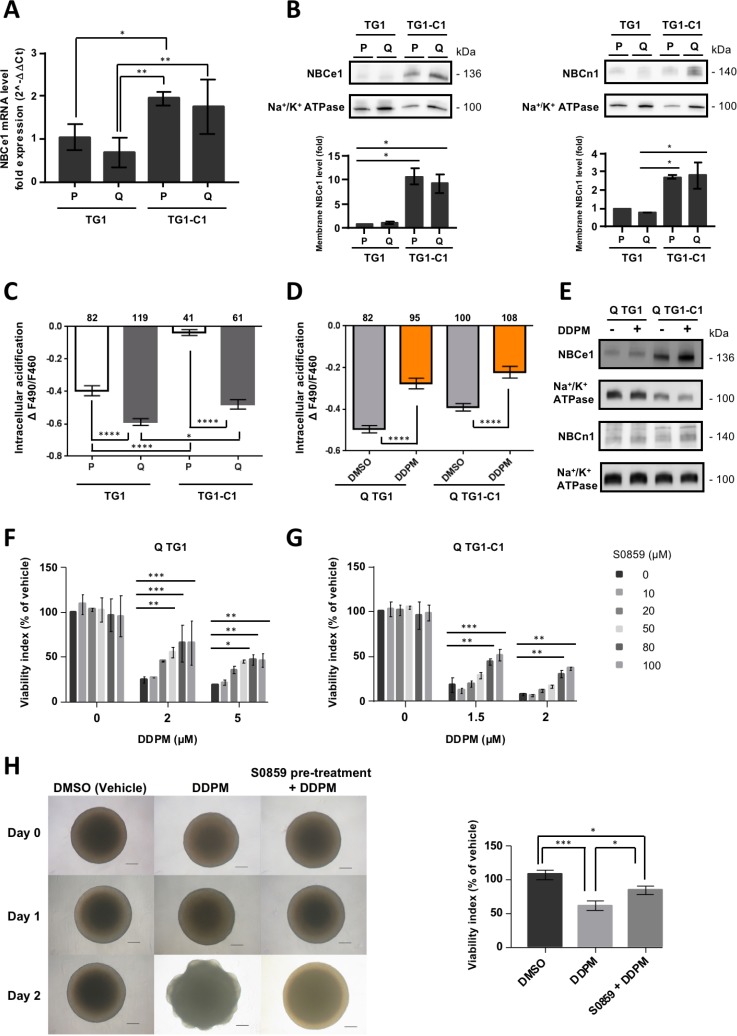
DDPM induces quiescent GSC cell death by deregulating Na^+^/HCO3^−^ cotransporter cell surface expression and activity (**A**) The mRNA expression levels of NBCe1 were determined by RT-qPCR in proliferating and quiescent TG1 and TG1-C1 GSC. 18S mRNA was used for normalization. Results were expressed as fold change (2^^-∆∆Ct^) considering proliferating TG1 GSC as calibrator. Data are from two independent experiments, each performed in duplicate (± SEM). Ordinary one-way ANOVA and Dunnett’s multiple comparisons test. ^*^p < 0.05, ^**^p < 0.005. (**B**) Membrane compartment NBCe1 (left panel) and NBCn1 (right panel) protein levels were evaluated in proliferating and quiescent TG1 and TG1-C1 GSC with commercially available antibodies. Na^+^/K^+^ ATPase levels in membrane protein extracts were used as loading controls. Relative membrane NBCe1 or NBCn1 levels (corrected to Na^+^/K^+^ ATPase) were represented as fold difference considering proliferating TG1 GSC as a calibrator. Quantitative results are from two independent experiments (± SEM). Ordinary one-way ANOVA and Dunnett’s multiple comparisons test. ^*^p < 0.05. (**C**) pH-dependent BCECF fluorescence intensity ratio F490/F460 reflecting amplitude of acidification upon perfusion of proliferating (P) or quiescent (Q) TG1 and TG1-C1 cells with a CO_2_/HCO3^−^ buffered saline solution. Data are from two independent cultures (± SEM). Numbers of cells are indicated above the plots. Ordinary one-way ANOVA and Tukey’s multiple comparisons test. ^*^p < 0.05, ^****^p < 0.0001. (**D**) Quiescent TG1 and TG1-C1 cells were treated with DDPM (10 µM, 2 hours in 0.1% DMSO used as vehicle). Control cells were incubated only in the presence of 0.1% DMSO. At the end of treatment, HCO3^−^ transport activity was measured as described in (C). Data are from two independent cultures (± SEM). Numbers of cells are indicated above the plots. Ordinary one-way ANOVA and Tukey’s multiple comparisons test. ^****^p < 0.0001. (**E**) Surface membrane levels of NBCe1 and NBCn1 were determined by Western blotting in quiescent (Q) TG1 and TG1-C1 GSC treated for 2 hours with DDPM (10 µM in 0.1% DMSO used as vehicle) or in DMSO alone (0.1%). Na^+^/K^+^ ATPase levels in surface membrane protein extracts were used as loading controls. Results for NBCe1 or NBCn1 are from samples loaded on nonconsecutive lanes of the same gel. (**F**, **G**) Quiescent TG1 (F) and TG1-C1 (G) GSC were pre-treated for 24 hours with increasing concentrations of the NBC-family Na^+^/HCO3^−^ cotransporter inhibitor S0859. Subsequently, cells were mock-treated (1% DMSO and S0859 from the pre-treatment) or incubated in the presence of DDPM (2 or 5 µM for TG1 GSC; 1.5 and 2 µM for TG1-C1 cells and S0859 from the pre-treatment) for 24 hours. Cell viability measurements were performed with the ATP-Glo cell survival-based assay. Results are expressed as a percentage of the viability of control TG1 or TG1-C1 GSC incubated with 1% DMSO alone for 48 hours. Results are the mean of two independent experiments performed in triplicate (± SEM). Statistical analysis was performed with Two-way ANOVA and Tukey’s multiple comparison test. ^*^p < 0.05, ^**^p < 0.005, ^***^p < 0.0005. (**H**) At day 0, TG1 GSC-derived macro-spheres were treated with DMSO alone (1%; upper left and middle panels) or with the NBC inhibitor S0859 (100 µM) in 1% DMSO (upper right panel) for 24 hours. DDPM (2 µM) was subsequently added at day 1 to macro-spheres (middle and right middle panels) and cells were incubated in the presence of the compound for another 24 hours. Scale bars: 200 µm. The ATP-Glo assay was used to evaluate cell viability which was represented as a percentage of the viability of the cells within the sphere treated in control conditions (1% DMSO, 48 hours). Results are from a distinct neurosphere for each condition tested in triplicate (± s.d.). Similar results were obtained in at least two independent additional experiments. Ordinary one-way ANOVA and Tukey’s multiple comparison test. ^*^p < 0.05, ^***^p < 0.0005.

To test this hypothesis, we first investigated the mRNA levels of NBCe1 by RT-qPCR, as well as the total membrane-bound NBCe1 protein levels by Western blotting total membrane protein extracts of proliferating and quiescent TG1 and TG1-C1 GSC (Figure 6A and 6B, respectively). Overall NBCe1 mRNA levels were higher in TG1-C1 cells compared to TG1 GSC, whereas no significant differences were seen between quiescent and proliferating GSC in either cell type (Figure 6A). Similarly, membrane-bound NBCe1 protein levels were significantly higher in TG1-C1 compared to TG1 GSC, but again no differences were detected between quiescent and proliferating GSC (Figure 6B, left panel). Total membrane NBCn1 levels were also found to be higher in proliferating and quiescent TG1-C1 GSC compared to TG1 cells (Figure 6B, right panel).

HCO3^−^ transport activity was then measured using intracellular pH imaging with BCECF in proliferating and quiescent TG1 and TG1-C1 cells. When bathing solution is switched from a HEPES-buffered solution to a solution buffered by 5% CO_2_/ 26 mM HCO3^−^, changes in intracellular proton concentration ([H+]I) are attributable primarily to CO_2_ diffusion across the cell membrane, conversion of CO_2_ to H^+^ and HCO3^−^, and subsequent intracellular acidification. Then, this acidification is regulated by HCO3^−^ transport [36, 37]. In agreement with higher overall NBCe1 and NBCn1 protein levels detected in TG1-C1 GSC, NBC cotransport activity was higher both in proliferating and quiescent TG1-C1 cells compared to the TG1 cell type since a lower amplitude of acidification was observed upon perfusion of these cells with the CO_2_/HCO3^−^ buffered saline solution (Figure 6C). Interestingly, HCO3^−^ transport activity was significantly lower both in quiescent TG1 and TG1-C1 cells compared to their proliferating counterparts pointing to the importance of controlling NBC cotransporter function for the maintenance of viable quiescent GSC (Figure 6C).

To test whether DDPM affects the steady state of NBC cotransport activity in quiescent GSC, intracellular pH imaging experiments were performed in quiescent TG1 and TG1-C1 cells following a 2-hour treatment with vehicle (DMSO) or with 10 µM of DDPM. As shown in Figure 6D, DDPM treatment resulted in an increase in HCO3^−^ transport activity in both types of GSC supporting the idea that DDPM causes quiescent GSC necrotic cell death by deregulating NBC cotransporter function. Moreover, as shown by Western blotting on biotinylated cell surface protein extracts prepared from quiescent TG1 and TG1-C1 cells, abnormally elevated HCO3^−^ activity in the presence of DDPM is associated to an increase in NBCe1 and NBCn1 cell surface levels (Figure 6E).

To further confirm the link between DDPM cytotoxicity and abnormal NBC cotransporter function, quiescent TG1 and TG1-C1 cells were pretreated for 24 hours with various concentrations of S0859, a NBC Na^+^/HCO3^−^ inhibitor that affects both electrogenic and electroneutral NBC-family cotransporters, including NBCe1 and NBCn1 [38, 39]. DDPM was then added for an additional time lapse of 24 hours. As expected, S0859 partially reversed the cytotoxic effect of DDPM in quiescent TG1 and TG1-C1 GSC (Figure 6F and 6G). This protective effect was observed in TG1 cells between 20 and 100 µM of S0859, and was statistically significant at concentrations ≥50 µM (Figure 6F). In quiescent TG1-C1 cells, higher concentrations (≥80 µM) were required to obtain statistically significant differences (Figure 6G). Lower expression levels of NBCe1 and NBCn1 and lower NBC cotransport activity in TG1 GSC (Figure 6A–6C) may explain why S0859 has a more protective effect on DDPM activity in these cells compared to TG1-C1 GSC.

Dose-response curves of DDPM with increasing concentrations of S0859 for quiescent TG1 and TG1-C1 GSC indicated that the IC50 values of DDPM are higher in the presence of S0859, whereas its maximal effect is reduced (Supplementary Figure 8A and 8B, respectively). These data indicate that DDPM and S0859 are probably not competing for the same binding sites on target proteins.

To distinguish between NBCe1 and NBCn1 contributions to the protective effect of S0859 on quiescent GSC, we used DIDS (4,4’-Diisothiocyanatostilbene-2,2’-disulfonate), a non-selective inhibitor of anion transport stilbene derivative that blocks NBCe1 and all other Na^+^-coupled HCO3^−^ cotransporters, but not NBCn1. Blockade of NBCn1 is poor even at concentrations of 500 µM of DIDS [34]. The effect of DIDS was assessed on quiescent GSC treated with DDPM. As shown in Supplementary Figure 9, TG1 GSC were highly sensitive to DIDS even in the absence of DDPM. TG1-C1 cells were less sensitive to the compound since higher concentrations of DIDS were required to induce cell death (compare graphs in left and right panels in Supplementary Figure 9). This differential sensitivity is in agreement with the higher expression levels of, at least, NBCe1 in these cells compared to TG1 GSC (Figure 6A and 6B). However, the effects of DDPM were not even partially reversed in the presence of DIDS (Supplementary Figure 9 left and right panels). Thus, either NBCn1 is not involved in DDPM cytotoxicity on GSC or the cytotoxicity of DIDS alone, namely on quiescent TG1 GSC, masks any protective effect.

The protective effect of S0859 was further assessed on TG1 GSC-derived macro-spheres. Pretreatment of such neurospheres with S0859 (100 µM) was again able to partially preserve TG1 GSC from DDPM cytotoxicity (Figure 6H, left and right panels). Altogether these data suggest that Na^+^/HCO3^−^ cotransporter cell surface expression and activity deregulation is mediating DDPM cytotoxicity on quiescent glioblastoma GSC.

### DDPM-induced WNK1 function deregulation acts downstream of Akt/SGK1 signaling on NBC cotransporter function in quiescent GSC

**Figure 7:**
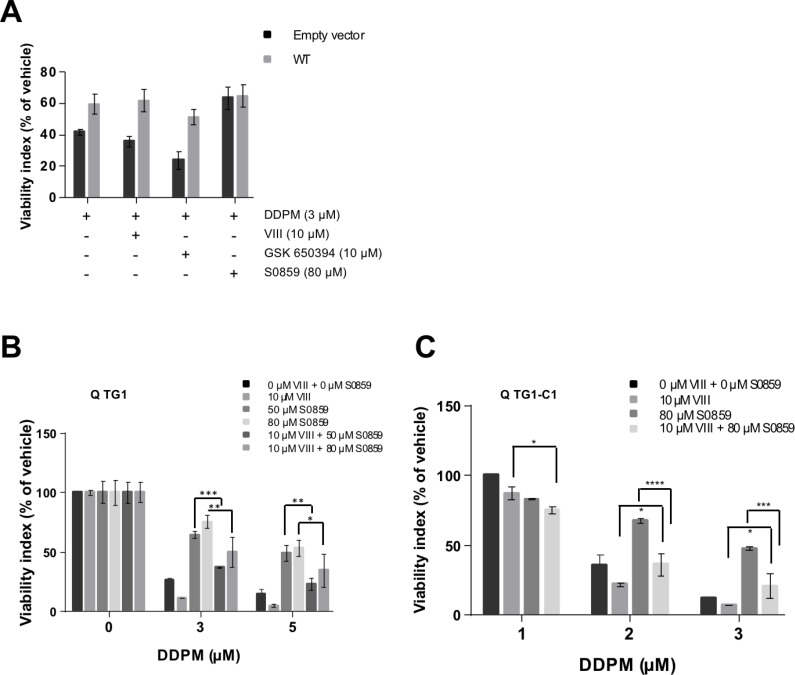
DDPM-induced WNK1 function deregulation acts downstream of Akt/SGK1 signaling on NBC cotransporter function in quiescent GSC (**A**) 3D macro-spheres were obtained from control TG1 GSC (Empty vector; dark grey bars) or TG1 GSC stably overexpressing wild-type (WT) WNK1 (light grey bars) and incubated in the presence of DMSO alone (1%) or pretreated with the indicated concentration of NBC inhibitor S0859 for 24 hours. DDPM alone (in 1% DMSO) or DDPM combined to 10 µM of the VIII Akt inhibitor or 10 µM of the SGK1 inhibitor (GSK 650394) were then added and treatment was pursued for 24 hours. Control cells were maintained in 1% DMSO. Cell viability was evaluated following treatment with the 3D ATP-Glo assay and expressed (as a percentage) for each type of sphere with respect to DMSO alone-treated controls. Two-way ANOVA, Empty vector versus WT WNK1: *p* = 0.0038 (^**^). (**B**, **C**) Mechanically dissociated quiescent TG1 (B) or TG1-C1 (C) GSC were incubated in 1% DMSO (controls) or pretreated for 24 hours with the indicated concentrations of the NBC inhibitor S0859 (in 1% DMSO). Indicated concentrations of DDPM alone (in 1% DMSO) or DDPM combined to 10 µM of the VIII Akt inhibitor were then added and treatment was pursued for 24 hours. Control cells were maintained in 1% DMSO. Cell viability measurements were performed with the ATP-based CellTiter-Glo^®^ cell viability assay. Results are expressed as a percentage of the viability of control GSC incubated with 1% DMSO alone (vehicle) for 24 hours. Results are the mean of two independent experiments performed in triplicate (± SEM). Statistical analysis was performed with two-way ANOVA and Tukey’s multiple comparisons test. ^*^p < 0.05, ^**^p < 0.005, ^***^p < 0.0005, ^****^p < 0.0001.

Results presented in previous sections argue in favor of the involvement of an Akt/SGK1-WNK1-NBC cotransporter signaling axis deregulation in DDPM cytotoxicity in quiescent GSC. In order to determine the level of action of WNK1 within this signaling axis, we first compared DDPM activity in Empty-vector and WT WNK1 overexpressing TG1 cell-derived macro-spheres in the presence of Akt, SGK1 and NBC cotransporter inhibitors. As shown in Figure 7A, Akt or SGK1 inhibition does not interfere with the protective effect of WT WNK1 overexpression in macro-spheres derived from GSC. These results suggest that WNK1 mediates DDPM cytotoxicity downstream of these kinases. In contrast, no protective effect of the NBC inhibitor S0859 is observed in macro-spheres stably overexpressing the WT WNK1 protein compared to control GSC (Figure 7A). This result is in favor of a role for NBC cotransporters downstream from WNK1 in the signaling pathway of DDPM in GSC.

Finally, as shown in both TG1 and TG1-C1 GSC, Akt inhibition by VIII partially reverses the protective effect of NBC inhibitor S0859 pre-treatment on quiescent cells treated with DDPM, suggesting a link between Akt and NBC Na^+^/HCO3^−^ cotransporter function and DDPM activity in these cells (Figure 7B and 7C, respectively).

## DISCUSSION

On account of their activity profile towards GSC, Bisacodyl and its pharmacologically active derivative, DDPM, are relevant to the development of novel, more effective treatments against glioblastoma. Clinically, Bisacodyl is currently used as a stimulant laxative to treat constipation and for bowel cleansing prior to colonoscopy. The laxative effect of the compound is attributed to effects on intestinal motility, water secretion or absorption and electrolyte homeostasis through inhibition of Na^+^/K^+^ ATPase, PKC-dependent stimulation of prostaglandin E2 release resulting in decreased aquaporin-3 expression in the colon, and inducible nitric oxide (NO) synthase stimulation and NO production in intestinal epithelia [40–43]. In quiescent GSC, we previously reported that this compound interferes with inositol 1,4,5-triphosphate receptor (IP3R1)-mediated intracellular calcium release induced by acetylcholine and carbachol [19].

Here, the phosphorylation of WNK1 T60 is demonstrated to decrease only in quiescent GSC treated with DDPM, and not in control conditions, indicating a probable relevance to the compound’s activity. Interestingly, basal p-T60 WNK1 levels were significantly higher in quiescent compared to proliferating TG1 and TG1-C1 GSC further supporting the involvement of WNK1 T60 phosphorylation levels in the maintenance of a viable quiescent GSC state.

Like T60 phosphorylation levels, the catalytic activity of the WNK1 protein was higher in quiescent compared to proliferating GSC in basal conditions. Treatment with DDPM resulted in a transient decrease of WNK1 catalytic activity in quiescent GSC, but this reduction appeared even more pronounced in non-responding cells maintained in proliferating culture conditions. Lower basal catalytic activation of WNK1 in proliferating GSC may contribute to the higher amplitudes of effects observed under such conditions.

Direct involvement of WNK1 protein function in DDPM activity on GSC is suggested by the observation that the compound’s cytotoxicity is reduced in macro-spheres derived from GSC stably overexpressing the wild-type human WNK1 isoform that is predicted to overcome reduction of p-T60 WNK1 levels and catalytic activity induced by this compound. The partial, but significant, reversal of DDPM cytotoxicity in GSC through overexpression of the phosphomimetic WNK1 mutants (T60D/E), but not of the non-phosphorylable (T60A) mutant, confirms that the phosphorylation status of WNK1 on T60 is definitely involved in the DDPM signaling pathway in GSC. Unexpectedly, T60A WNK1 overexpression did not decrease but rather weakly favored cell viability upon DDPM treatment suggesting that other WNK1 functions may be involved in the compound’s effect on GSC. In agreement with this observation, overexpression of a kinase-dead mutant of the protein did not preserve GSC from DDPM activity suggesting that the inhibition of the protein’s catalytic activity in quiescent GSC treated with DDPM, would also be required for the compound’s effect. If this is the case, catalytically constitutively active mutants of WNK1, reversing the effect of the compound on WNK1 enzymatic activity, would be expected to show a protective effect on GSC.

WNK1 T60 phosphorylation and functions can be modulated by the protein kinases Akt and SGK1 in other cell systems [32, 33]. Even though Akt phosphorylation at its C-terminus has been associated to cell cycle progression [44], a negative role of Akt activation on regulated mitogenesis was reported in some cells [33] and higher levels of the phosphorylated, presumably active forms of Akt and SGK1 were detected in quiescent GSC, in agreement with the observations for WNK1. Moreover, DDPM treatment resulted in a significant activity-dependent reduction in the phosphorylation/activation status of these proteins. Inhibition of Akt and/or SGK1 resulted in a decrease in WNK1 T60 phosphorylation suggesting that DDPM may target quiescent GSC through an Akt and/or SGK1-dependent reduction of the phosphorylation status of WNK1 on this residue.

**Figure 8:**
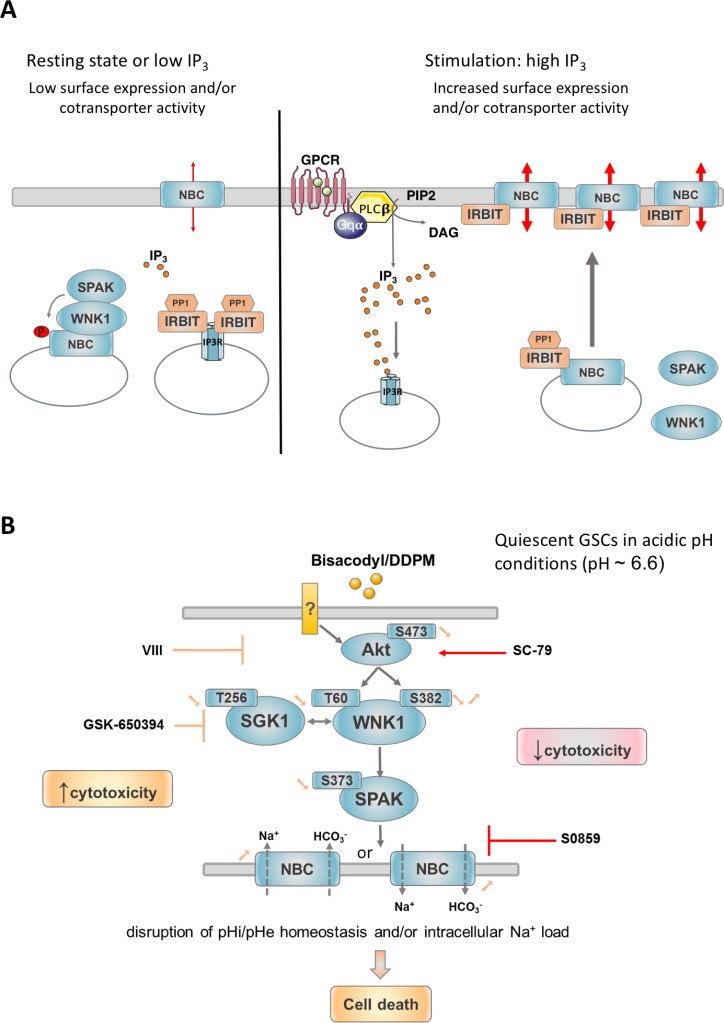
Mechanisms proposed to regulate NBC cotransporter surface expression and activity and proposed model for signaling pathways underlying the cytotoxicity of DDPM in GSC (**A**) In resting conditions or when IP_3_ levels are low, IRBIT (IP_3_R binding protein released with inositol 1,4,5-triphosphate) is bound to IP_3_ receptors (IP_3_R) and suppresses their activation. WNK1 recruits SPAK on NBC cotransporters. NBC N-terminal phosphorylation by SPAK sequestrates these proteins in intracellular vesicles. Upon stimulation producing high IP_3_ levels, for example through G protein coupled receptor (GPCR) activation, IP_3_ displaces IRBIT from IP_3_R. IRBIT is thus able to bind to NBC cotransporters present in intracellular vesicles and to recruit PP1 (Protein phosphatase 1) leading to NBC dephosphorylation and recruitment to the plasma membrane. IRBIT is also able to directly positively modulate plasma membrane bound NBC cotransporter activity presumably by binding to and partly masking their N-terminal autoinhibitory domain. PIP_2_: phosphatidylinositol 4,5-bisphosphate; DAG: diacylglycerol; PLCβ: phospholipase C β; Gqα: Gq protein α subunit. Adapted from [56]. (**B**) In quiescent GSC maintained in a slightly acidic environment, the active metabolite of Bisacodyl, DDPM, was shown to negatively regulate the phosphorylation status of WNK1 T60, which is potentially phosphorylated by kinases Akt and SGK1. The catalytic activity of WNK1 requiring phosphorylation of S382 is also transiently reduced by the compound. This would lead to reduced SPAK activity which in turn was shown to result in increased cell surface expression and activity of NBC Na^+^/HCO3^−^ cotransporters including NBCe1 and NBCn1. NBCe1 and NBCn1 are able to regulate intracellular pH homeostasis (pHi alcalinization) through HCO3^−^ influx. According to Na^+^: HCO3^−^ stoichiometry or gradient reversal that may occur even in slightly acidic pH conditions, NBCe1 can also operate net efflux of HCO3^−^ with opposite effects on intracellular and extracellular (pHe) pH regulation. Stoichiometry of Na^+^ and HCO3^−^ co-transport by NBC transporters is not indicated. Only the direction of co-transport is shown. Deregulation of pHi/pHe homeostasis outside of cell survival ranges may underlie the effect of the compound on GSC (necrosis). Alternatively, Bisacodyl/DDPM cytotoxicity may result from intracellular Na^+^ overload (co-transported with HCO3^−^) which was shown to lead to abnormal intracellular calcium concentrations, mitochondrial damage and necrotic cell death. In agreement with these findings, Akt and SGK1 inhibitors reduce WNK1 T60 phosphorylation levels and reinforce the activity of Bisacodyl/DDPM on quiescent GSC. Conversely, Akt activation or NBC inhibition produce a protective effect. ? indicates that direct protein targets of Bisacodyl/DDPM in GSC are currently unknown.

The N-terminal domain of WNK1 containing T60 has previously been shown to act as a scaffold that recruits the SPAK protein kinase to the N-terminal domain of the Na^+^/HCO3^−^ cotransporter NBCe1. This WNK1 function, which is independent of its catalytic activity, results in the phosphorylation of the N-terminal domain of NBCe1 by SPAK and has an inhibitory effect on the cotransporter by reducing its cell surface expression. This is antagonized by IRBIT (inositol receptor binding protein released with inositol 1, 4, 5-triphosphate), an enzyme which regulates both inositol 1, 4, 5-triphosphate receptors and ion cotransporters including NBCe1 [36]. At the resting state or when inositol 1, 4, 5-triphosphate (IP3) concentrations are low near IP3 receptors, IRBIT binds to IP3 receptors and suppresses their activation. Increased levels of IP3 displace IRBIT and activate IP3R-mediated Ca^2+^ release from intracellular stores. Released IRBIT can then bind to NBC cotransporters and enhance their activity both by increasing their cell surface levels and by directly modulating their activation status (Figure 8A). Interestingly, higher expression of IP3R1 was seen in quiescent GSC under acidic conditions [19]. Thus, in these cells, the binding of IRBIT to IP3 receptors combined with high levels of WNK1 T60 phosphorylation may cooperate to maintain NBC cotransporter activity to levels allowing cell survival in quiescent culture conditions. Other cotransporters, including NBCn1, share similar N-terminal domains with NBCe1 and could thus be regulated by similar mechanisms [36]. It was thus conceivable, that DDPM, by reducing p-T60 levels in the N-terminus of WNK1, relieves the regulatory effect of WNK1 on NBCe1 and/or other related cotransporter-cell surface expression level and function, and that the excessive activation of these proteins leads to necrotic death. Data presented in this study confirm this hypothesis since differences in HCO3^−^ transport activity were measured between proliferating and quiescent GSC with lower activity levels present in quiescent cells. Moreover, DDPM treatment of quiescent cells resulted in a significant increase in NBC cotransporter function associated to an increase in cell surface levels of NBCe1 and, to a lesser extent, NBCn1. A combined treatment of DDPM with S0859, an inhibitor of NBC Na^+^/HCO3^−^ cotransporters including NBCe1 and NBCn1, significantly reduced the cytotoxic effect of the compound. This result implies that the DDPM effect on Na^+^/CO3^−^ cotransporter function is responsible for its cytotoxicity. Interestingly, NBCe1 mRNA levels, membrane protein expression levels of both NBCe1 and NBCn1 and NBC cotransporter activity are higher in TG1-C1 GSC compared to TG1 cells. These higher expression levels and activity may explain the higher sensitivity of TG1-C1 cells to DDPM [20] and why the Na^+^/HCO3^−^ cotransporter inhibitor S0859 is less effective at protecting these cells. Unfortunately, it was not possible to determine the respective contribution of these cotransporters to Bisacodyl/DDPM cytotoxicity using DIDS, a compound that inhibits several cotransporters including NBCe1, but not NBCn1, because DIDS alone was also cytotoxic.

The involvement of Akt and SGK1 kinases in DDPM activity in GSC was further supported by the fact that modulation of these kinases using targeting inhibitors (for Akt and/or SGK1) or activators (for Akt) influenced the compound’s effect on GSC. Furthermore, a functional link between at least Akt and Na^+^/HCO3^−^ cotransporters of the NBC family potentially regulated by WNK1/SPAK was established. As expected by our hypothesis, Akt inhibition which would increase Na^+^/HCO3^−^ cotransporter activity leading to cell death, partially reversed the protective effects of the S0859 Na^+^/HCO3^−^ cotransporter inhibitor. Again, differences in Na^+^/HCO3^−^ cotransporter expression levels and activity may underlie the less pronounced effects of these modulators on TG1-C1 compared to TG1 GSC.

Based on our experimental data, we propose that DDPM acts on quiescent GSC under extracellular acidic conditions and on the inner-layer slow-growing cells exposed to hypoxic/acidic microenvironments in GSC-derived macro-spheres by down regulating the Akt-SGK1-WNK1 pathway that leads to a non-physiological increase in plasma membrane Na^+^/HCO3^−^ cotransporter levels and function and eventual necrotic cell death of GSC (Figure 8B). It is of note, that although no differences in NBCe1/NBCn1 Na^+^/HCO3^−^ cotransporter total membrane expression levels are found between DDPM-sensitive quiescent GSC and non-responding proliferating GSC maintained in physiological pH conditions, NBC cotranporter activity is significantly lower in quiescent GSC. Thus, preferential targeting of these cells by DDPM is probably linked to their greater dependence on cotransporter function compared to proliferating cells unchallenged by growth factor deprivation and acidic stress.

To avoid cell death in acidic extracellular conditions, intracellular pH (pHi) has to be maintained at slightly alkaline values (pH 7.2-7.4). Tumor cells have thus developed various mechanisms to maintain an alkaline pHi and a slightly acidic extracellular pH (pHe). For example, these cells have increased ability to export acidic catabolites (such as carbon dioxide, carbonic acid or lactic acid) and import weak bases (such as HCO3^−^ ions) using transporters. In addition, H^+^ ions are directly extruded by exchange for other cations or by vacuolar ATPase (V-ATPase) [45]. NBCe1 and NBCn1 Na^+^/HCO3^−^ cotransporters catalyze HCO3^−^ influx which would subsequently lead to pHi alcalinization and extracellular pH (pHe) acidification. Depending on Na^+^:HCO3^−^ stoichiometry (2 or 3 HCO3^−^ for 1 Na^+^) or gradient reversal even in slightly acidic conditions, the electrogenic NBCe1 cotransporter can also operate net efflux of HCO3^−^ with opposite effects on pHi/pHe regulation [46]. Thus, deregulation of Na^+^/HCO3^−^ cotransporters operated by DDPM may disrupt intracellular and/or extracellular pH homeostasis outside of the ranges required for cell survival and thereby lead to cell death. Alternatively, necrotic cell death in GSC treated with DDPM may result from an abnormal modification of intracellular Na^+^ which is concomitantly transported by NBCe1/NBCn1 proteins. Elevated intracellular Na^+^ has been shown to result in intracellular Ca^2+^ overload leading to mitochondrial damage and necrotic cell death. Moreover, NBC cotransporters have been reported to modify intracellular Na^+^ concentration modifications in several systems [47, 48]. Altogether, our data strongly support the idea that deregulation of an Akt/SGK1/WNK1/NBC cotransporter-related pathway is involved in Bisacodyl/DDPM cytotoxicity in GSC, although the direct protein targets of this compound in these cells remain to be identified. Interestingly, Bisacodyl/DDPM shows activity and similar effects in deregulation of WNK1 and its signaling partners on two biologically distinct GSC types derived from patients, TG1 and TG1-C1 cells, suggesting that the effect of the compound is not specific to a single GSC cell type. In view of the high inter- and intra-tumoral heterogeneity of glioblastomas [49], this property enhances the potential for the use of the compound as a new anticancer drug. Specific characteristics of TG1 and TG1-C1 cells, *i.e.* higher expression levels and activity of NBC cotransporters in TG1-C1 cells, may explain the variability in the amplitude of responses observed in some cases further supporting the involvement of the Akt/SGK1/WNK1/NBC cotransporter axis in DDPM cytotoxicity.

In a previous report, we showed that pretreatment with DDPM inhibits Ca^2+^ mobilization from intracellular stores mediated by acetylcholine or carbachol IP3 (inositol 1,4,5-triphosphate) production and activation of IP3 receptors including IP3R1 [19]. Interestingly, Akt -mediated phosphorylation of WNK1 T60 was shown to promote PIP2 (phosphatidylinositol 4,5-bisphosphate) synthesis through phosphatidylinositol 4-kinase IIIα activation, thus sustaining PLC-β signaling, IP3 production and IP3 receptor activation mediated by Gq-coupled GPCRs (G protein coupled receptors) [50]. Inhibition of WNK1 T60 phosphorylation elicited by DDPM may thus underlie the negative effect of the compound on Ca^2+^ signaling in quiescent GSC [19].

To date, several WNK1 mutations have been associated with cancer although their contribution to tumor initiation is unknown [51]. Furthermore, WNK1 functions have been associated with cell proliferation, migration and survival. Cell stress conditions, in particular metabolic stress have revealed that WNK1 protein is important for maintaining cells in a viable state [52]. In agreement, our observations that DDPM disrupts the WNK1 pathway, thereby provoking cell death in quiescent GSC, provide further evidence that WNK1 function regulating downstream transporters is a critical event for maintaining a viable quiescent state for cells challenged by specific microenvironmental conditions. Conversely, the WNK1 pathway does not seem to influence the viability of GSC maintained in proliferating conditions. By regulating water, electrolyte and pH homeostasis, as well as receptor trafficking (including glucose transporters) at the cell surface, WNK1 may also play a role in the metabolic adaptation of cancer cells to their microenvironment [53, 54]. Deregulation of these processes may underlie the effects of WNK1 mutations in cancer. In glioblastoma, WNK1 was involved both in resistance to TMZ-induced apoptosis and cell migration by controlling the NKCC1 cotransporter [26], but none of these studies focused on cancer cells with stem-like properties. To our knowledge, this is the first study directly implicating the WNK1 protein signaling in the maintenance of viable cancer stem-like cells isolated from glioblastoma, thus uncovering novel, potential therapeutic targets for the treatment of this disease.

## MATERIALS AND METHODS

### Materials

Bisacodyl (4,4’-diacetoxydiphenyl-2-pyridyl-methane; CAS number: 603-50-9) was purchased from Sigma-Aldrich. DDPM (4,4’-dihydroxydiphenyl-2-pyridyl-methane), the active derivative of Bisacodyl was synthesized in-house based on previously described methods [20]. Methods for the synthesis of the inactive derivative of Bisacodyl, DDPM, are provided in Supplementary Materials and Methods. Chemical structure of the DDPM inactive derivative and activity profile of the compound on quiescent TG1 GSC are shown in Supplementary Figure 2.

S0859 NBC cotransporter inhibitor (CAS 1019331-10-2), DIDS (4,4’-Diisothiocyanatostilbene-2,2’-disulfonate), a stilbene derivative that can inhibit NBCe1 and all other Na^+^-coupled HCO3^−^ transporters but not NBCn1 (CAS 207233-90-7), Akt activator SC-79 (CAS 305834-79-1) and SGK1 inhibitor GSK 650394 (CAS 890842-28-1) were purchased from Sigma-Aldrich. The Akt inhibitor VIII (CAS 612847-09-3) was from Calbiochem (Merck Millipore).

### Ethics statement

The biomedical research was conducted according to the declaration of Helsinki, to the French laws and was approved by the institutional review board of Sainte Anne Hospital, Paris, France. Patients have given written informed consent.

### Primary glioblastoma stem cell culture

TG1 and TG1-C1 (OB1) glioblastoma (WHO grade IV glioma) stem-like cells (GSC) were derived from tumor samples of patients (Sainte Anne Hospital, Paris, France) as previously described [20] and expanded as neurosphere cultures in NS34 medium. In proliferating cultures, neurospheres were mechanically dissociated in single-cell suspensions twice a week. Quiescent cells were obtained by non-renewal of the medium for 9 days following mechanical dissociation and cell seeding in NS34 medium. The quiescent slow-growing state of cells obtained in these conditions as well as its reversibility were verified [20]. Both proliferating and quiescent GSC were also phenotypically and functionally characterized as to the expression of stemness and pluripotency markers as well as to their clonal, *in vitro* differentiation and *in vivo* engraftment properties [20]. Master and working cell banks were established for all cell types. Cells were used at defined ranges of cell passages.

### *In vitro* formation of clonal and non-clonal macro-spheres from GSC

For clonal-macro-sphere formation, GSC were dissociated into single cells and seeded into 96-well plates at a density of 1 cell/well in 200 μL of NS34 medium. 50 μL of medium were added into each well every week and individual spheres were transferred into 24-well plates in 1 mL of NS34 medium at week 2. One single sphere was kept in each well to avoid sphere aggregation. The medium was changed once a week. For non-clonal macro-spheres, GSC were dissociated and seeded into 96-well round bottom plates at a density of 5000 cells/well in 200 µL of NS34 medium. The plates then were centrifuged at a speed of 1000 rpm for 5 min to precipitate the cells at the bottom of well. The medium was changed once a week during the first 2 weeks and every 2 days afterwards. Individual spheres were used when they reached a size of 800 µm to 1 mm of diameter for treatments with vehicle (DMSO), DDPM or DDPM combined to NBC, Akt and or SGK1 inhibitors as indicated.

### Construction of plasmids for WT and mutant WNK1 overexpression

Full length synthetic gene for human WNK1 (GenBank accession No. NM_018979.3) assembled from oligonucleotides and/or PCR products and cloned into a pMA plasmid was purchased from GeneArt. WNK1 coding sequence was PCR amplified with primers including attB1 and attB2 sites for Gateway cloning (Thermo Fisher Scientific). AttB1-WNK1-attB2 amplified fragments were gel-purified (Macherey-Nagel NucleoSpin Gel and PCR clean-up kit). The WT WNK1 coding sequence was then introduced *via* a BP recombination reaction into the pDONR 207 vector. Point mutations to obtain T60A, T60/D, E and K233M mutants were generated in WNK1 coding sequences in the pDONR 207 vector with the QuickChange II XL Site-Directed Mutagenesis kit following the instructions of the manufacturer (Agilent Technologies). All constructs were verified by sequencing of the entire open reading frames and surrounding sequences. A LR recombination reaction was then performed based on standard Gateway cloning protocols to obtain pCIneo 3-FLAG Gateway system mammalian expression vectors for overexpression of WT and mutant isoforms of WNK1 in GSC. Sequences of the entire cloned fragments and surrounding vector were also verified. A pCIneo 3-FLAG control vector with several STOP codons immediately downstream of the 3-FLAG tag encoding sequences (Empty vector) was used as a control. Gateway system materials and vectors were kindly provided by Dr Réal (Faculty of Pharmacy, Strasbourg, France). Primer sequences used for PCR amplification and cloning, mutagenesis and sequencing are available in Supplementary Table 1.

### Procedure for establishing TG1 GSC overexpressing WT and mutant forms of WNK1

TG1 GSC were transfected with the pCIneo-3FLAG STOP control vector (empty vector) or with pCIneo-3FLAG vectors encoding WT and mutant (T60A, T60D, T60E and K233M) WNK1 with Primary Cell Nucleofector™ Solution P3 and the Amaxa 4D-Nucleofector™ System (Lonza) according to the manufacturer’s instructions. Non-transfected TG1 GSC were used as controls. 48 hours post-transfection, antibiotic-containing fresh medium was used for the selection which was performed with geneticin G418 (Sigma Aldrich) at a concentration of 400 µg/mL for a week until the negative control cells were all dead. Antibiotic concentrations were then reduced to 200 µg/mL for two more weeks. At the end of the selection procedure, TG1 cells stably expressing WT and mutant forms of WNK1 and TG1 cells stably transfected with the pCIneo-3FLAG STOP vector (Empty vector) were maintained in medium with G418 (100 µg/mL) for subsequent experiments.

### Cell viability measurements

Following dissociation, cells were plated in 96-well micro-well plates (opaque bottom) (Greiner) in 100 µL of NS34 medium, at a density ranging from 30,000 to 50,000 cells/well and treated for 24 hours with DDPM and/or Akt and/or SGK1 inhibitors at indicated concentrations. For combinations of DDPM with NBC cotransporter inhibitor S0859, Akt activator SC-79 or DIDS, GSC were pre-treated with these compounds for 24 hours prior to addition of DDPM. Inhibitor and DDPM concentrations in each experiment are given in Figure legends. Negative control wells contained cells treated with DMSO (1%; vehicle). At the end of treatments, CellTiter-Glo^®^ luminescent cell viability assay was used according to the instructions of the manufacturer (Promega). Luminescence was measured in a multilabel reader, including a luminometer (EnVision™, PerkinElmer). Each condition was tested in triplicates.

For macro-spheres, cell viability assays were performed with CellTiter-Glo^®^3D cell viability assay (Promega). Spheres (one sphere per well), were plated in 100 μL of medium (1% DMSO) containing or not DDPM and/or inhibitors of Akt and SGK1. Pre-treatment with S0859 was performed as indicated in the corresponding figure legend. At the end of treatment (24 hours or longer as indicated), reagent of cell viability assay was added (100 μL) and after 30 minutes of incubation, 100 μL of mixed cell-reagent solution was taken from each well and measured with a luminometer as described above.

### Human phospho-kinase array

A human phospho-kinase array purchased from R&D Systems was used for profiling the relative site-specific phosphorylation in several kinases and kinase substrates, in quiescent GSC. TG1-C1 cells were incubated with DDPM (10 µM in 1% DMSO) for 2 hours or treated with vehicle (1% DMSO). Cell lysates were then prepared with RIPA lysis buffer system (Santa Cruz Biotechnology) (250 μL of complete RIPA buffer supplemented with PMSF, sodium orthovanadate and protease inhibitor cocktail (according to the supplier’s instructions) per 5 × 10^6^ cells). Total protein concentrations in extracts were determined with DC Protein Assay from Bio-Rad according to the microplate assay protocol of the manufacturer. Phosphokinase array membranes were then incubated with 200 µg of protein extract in each condition overnight at 4° C. Following a washing step with washing buffer provided with the kit, membranes were incubated with provided secondary antibodies at room temperature for 2 hours and then in the presence of a solution containing streptavidin coupled to horseradish peroxidase (HRP) at room temperature for 30 min. Luminescent signals obtained after incubation with revealing solution, were detected with the Image Quant LAS 4000 imaging system (GE Healthcare Life Science). Spots were quantified with the ImageJ software.

### Western blot analysis

For total cell protein extract preparation, dissociated proliferating or quiescent TG1 and TG1-C1 GSC treated with DDPM (10 µM in 1% DMSO, 10, 30 and 120 min) or 1% DMSO, quiescent GSC of both types treated with the inactive derivative of the compound (10 µM in 1% DMSO, 10, 30 and 120 min) or 1% DMSO as well as dissociated TG1 cells overexpressing or not (Empty vector), WT and mutant isoforms of WNK1 and quiescent TG1 and TG1-C1 GSC treated with VIII (10 µM for TG1 and 20 µM for TG1-C1 in 0.5% DMSO) and/or GSK 650394 (30 µM for TG1 and 80 µM for TG1-C1 in 0.5% DMSO), were collected and lysed in complete RIPA lysis buffer (Santa Cruz Biotechnology) and several passages through a 23G needle. Following an incubation of 20 min at 4° C, cell extracts were centrifuged for 15 min at 14000 rpm at 4° C. Cleared suspensions were quantified with the DC Protein Assay from Bio-Rad. Equivalent amounts of proteins (25–50 µg) were subsequently analyzed in 4–15% gradient Mini-PROTEAN TGX pre-cast gels from Bio-Rad and immunoblotting with appropriate antibodies listed in Supplementary Table 2. Specific antibody binding was detected by horseradish peroxidase-conjugated anti-rabbit or anti-mouse secondary antibodies. Proteins were visualized with the ECL Prime reagent (GE Healthcare Life Sciences) and the Image Quant LAS 4000 imaging system (GE Healthcare Life Science). Quantification of protein bands corrected to loading controls was done with the ImageJ software.

For extraction of total membrane proteins, cells were collected and lysed in a lysis buffer prepared with 10 mM HEPES pH 7.4 (Thermo Fisher Scientific) and 2 mM EGTA (Sigma), completed with 1x protease inhibitor cocktail (Roche). Several passages through a 23G needle were performed to break the cells. The post nuclear supernatant was obtained after centrifugation (3000 rpm) for 10 minutes at 4° C. Membrane protein extracts were recovered by centrifugation of the post-nuclear supernatant for 90 minutes at 14000 rpm at 4° C, resuspended in PBS and quantified with DC protein Assay (Bio-Rad).

Isolation of cell surface proteins was achieved through biotinylation followed by Neutravidin agarose affinity chromatography isolation of labeled proteins using the Pierce Cell Surface Protein Isolation Kit from Thermo Scientific according to the manufacturer’s instructions. Briefly, 40 × 10^6^ of quiescent (9 days post-medium renewal) TG1 and TG1-C1 cells were dissociated, resuspended in quiescent culture conditioned medium and treated for 2 hours at 37° C with DDPM (10 µM in 0.1% DMSO). Equal numbers of quiescent TG1 and TG1-C1 cells were incubated during the same period only in the presence of 0.1% DMSO and used as controls. Following treatment, cells were washed in ice-cold PBS and incubated for 30 min at 4° C in the presence of the membrane impermeable Sulfo-NHS-SS-Biotin labeling solution provided with the kit. Subsequent quenching of the reaction was followed by cell lysis in 500 μl of the provided lysis solution supplemented with 1× protease inhibitor cocktail from Roche. Biotinylated proteins were isolated with NeutrAvidin resin, eluted in 400 μl of Pierce^™^ Lane Marker Non-Reducing Sample Buffer (Thermo Scientific) containing 50 mM of DTT and analyzed by Western blotting (20 μl of extract per lane) using anti-NBCe1, NBCn1 and Na^+^/K^+^ ATPase antibodies (Supplementary Table 2).

### Intracellular pH imaging

Changes in pHi were measured using an imaging system associated with the use of an acetoxymethyl-ester form of the proton-sensitive dye BCECF-AM (Molecular probes, Grand Island, NY, USA) [37, 55]. Proliferating and quiescent TG1 and TG1-C1 cells were mechanically dissociated, resuspended in their respective media, and plated on glass coverslips previously coated with poly-D-lysine (0.02 mg/mL; Sigma, Lyon, France) for 30 min at 37° C. Cells were loaded with the proton-sensitive dye BCECF by incubating the cultures for 10 min at room temperature (22–25° C) in the extracellular solution containing 2.5 µM of the compound. Cells were washed three times with extracellular saline solution before and after loading. Before the loading step, quiescent TG1 and TG1-C1 cells were also treated with DDPM (2 h at 37° C in 0.1% DMSO) or with DMSO alone (0.1%).

During imaging experiments, cultured cells were continuously superfused with an HEPES-buffered extracellular saline solution containing: 140 mM NaCl, 5 mM KCl, 0.5 mM NaH2PO4, 2 mM CaCl2, 1 mM MgCl2, 10 mM glucose, and 10 mM Hepes (pH 7.4). Acidification and HCO3^−^ transport were initiated by perfusing the cells with HCO3^−^ buffered solution containing: 114 mM NaCl, 5 mM KCl, 0.5 mM NaH2PO4, 2 mM CaCl2, 1 mM MgCl2, 10 mM glucose, and 26 mM NaHCO3, continuously bubbled with 95% O_2_ and 5% CO_2_.

Fluorescence measurements on individual cells were performed with an inverted microscope (Axiovert 35; Zeiss, Gottingen, Germany) with an oil-immersion 940 objective (Fluor 40; NA, 1.30; Nikon, Tokyo, Japan), and a quantitative real-time imaging system comprising a cooled CCD camera (CoolSNAP HQ; Roper Scientific, Tucson, AZ, USA) and an image analysis software package (IMAGING WORKBENCH 4.0; Axon Instruments, Molecular Devices). Cells were alternately excited at wavelengths of 490 nm (proton-sensitive wavelength) and 460 nm (close to isosbestic point), and emitted light was collected above 510 nm. Pairs of images were acquired every 2.5 sec. Intracellular proton levels and their variations are expressed as the ratio of fluorescence signals (ratio F490/F460); The ratio F490/F460 changes inversely with the change in [H^+^]I and so directly reflects changes in pHi. This ratio was calculated after background signal subtraction. All experiments were performed at room temperature (22–25° C).

### RNA extraction and RT-qPCR

Total RNA was isolated from 5–10×10^6^ proliferating or quiescent (9 days without medium renewal) TG1 and TG1-C1 GSC using the TRI Reagent (Euromedex, France) according to the manufacturer’s instructions. RNeasy mini kit columns (Qiagen) were used for further purification of RNA samples. NanoDrop ND-1000 (Labtech) was used for absorption spectra analysis and RNA purity assessment. Absorption ratios A260/A280 and A260/A230 were comprised between 1.8 and 2.1. RNA concentration was determined using the Qubit fluorimeter and the Quant-it RNA Assay Kit (Invitrogen). RNA integrity was further evaluated with an Agilent 2100 Bioanalyzer and the RNA 6000 LabChip kit. Only RNA with a RNA Integrity Number (RIN) higher than 9 was processed (2100 expert software, Agilent Technologies). 1 µg of total RNA was reverse transcribed to single-stranded cDNA using the High Capacity cDNA Reverse Transcription kit (Applied Biosystems, Life Technologies). Real-time PCR analysis was performed with individual TaqMan gene expression assays in an ABI Prism 7000HT apparatus (Applied Biosystems, Life Technologies) using standard experimental conditions designed by the manufacturer. NBCe 1/SLC4A4 assay ID: Hs00186798-m1. Results were normalized to the 18S rRNA expression levels determined in all conditions (assay ID: 4319413E). Results are shown as mean ± SEM of two independent experiments performed in triplicates.

### Statistical analysis

Data were obtained from at least two independent experiments and are shown as mean (± SEM) or mean (± s.d.) as indicated in figure legends. *P* values were determined using unpaired two-tailed *t*-tests to compare two groups or one-way or two-way ANOVA to compare more than two groups. Indications are given in figure legends. The level of significance was set at *p* < 0.05. Statistical analyses were performed with the Prism 7.0 software (GraphPad).

## SUPPLEMENTARY MATERIALS FIGURES AND TABLES





## Abbreviations

GBM: glioblastoma
GPCR: G protein coupled receptor
GSC: glioblastoma stem-like cells
IP3: inositol 1, 4, 5-triphosphate
IRBIT: inositol receptor binding protein released with inositol 1, 4, 5-triphosphate
NBC: Na^+^/HCO3^−^ cotransporter
NO: nitric oxide
OSR1: oxidative stress-responsive kinase 1
PDK1: phosphoinositide-dependent protein kinase 1
PIP2: phosphatidylinositol 4,5-bisphosphate
SGK1: serum and glucocorticoid-stimulated protein kinase-1
SPAK: STE20/SPS1-related proline/alanine-rich kinase
TMZ: temozolomide
WNK1: with no lysine (K) kinase 1
