# Supplementary material for: WNK1 kinase and its partners Akt, SGK1 and NBC-family Na+/HCO3− cotransporters are potential therapeutic targets for glioblastoma stem-like cells linked to Bisacodyl signaling
Source: Oncotarget. 2018 Jun 5;9(43):27197–219. doi: 10.18632/oncotarget.25509 (PMC6007472; doi:10.18632/oncotarget.25509)
Supplement: Supplementary file 1 [file oncotarget-09-27197-s001.pdf]

WNK1 kinase and its partners Akt, SGK1 and NBC-family Na^+/ $HCO3^-$ cotransporters are potential therapeutic targets for glioblastoma stem-like cells linked to bisacodyl signaling

SUPPLEMENTARY MATERIALS

Synthesis of Bisacodyl/DDPM inactive derivative

The Bisacodyl/DDPM inactive derivative (LPI3271) was synthesized starting from 6-bromo-2-pyridinecarboxaldehyde and phenol through the chemical strategy described by Zeniou *et al.* [1]. LPI3271 (N-(6-aminohexyl)-6-{6-[bis(4-hydroxyphenyl)methyl]pyridin-2-yl}hexanamide as a trifluoroacetate salt): Anal. RP-HPLC purity > 98%. 1H NMR ($MeOD_4$): δ 1.38 (m, 6H), 1.50 (m, 2H), 1.65 (m, 4H), 1.75 (m, 2H), 2.19 (m, 2H), 2.91 (m, 2H), 3.05 (m, 2H), 3.15 (m, 2H), 5.86 (s, 1H), 6.80 (m, 4H), 6.93 (m, 4H), 7.38 (m, 1H), 7.78 (m, 1H), 8.38 (m, 1H); 13C NMR ($MeOD_4$): δ 26.54, 27.11, 27.46, 28.54, 29.65, 30.23, 30.35, 34.04, 36.79, 40.27, 40.85, 54.09, 116.99, 126.02, 126.37, 131.44, 131.57, 147.84, 158.36, 159.57, 160.39, 176.08. MS (ESI+): m/z $[M + H]^+$ calculated for $C_{30}H_{40}N_3O_3$, 490,3, found 490.2.

WNK1 shRNA-mediated knockdown

Human WNK1 cDNA fragments encoding the 19-nucleotide siRNA sequence 5′-CAATGAG TCAGATATCGAA-3′ derived from the target transcript or scramble siRNA sequence 5′-ACTACGAATGAC GTATAGA-3′, separated from their reverse 19-nucleotide complement by a short spacer, were cloned in the pEGFP-N2 RNAi vector kindly provided by Dr. N. Vitale (INCI, Strasbourg, France) downstream a H1-RNA promoter. Briefly, single-strand LIC sites (derived from the LIC cloning systems from Novagen) were added by asymmetrical PCR (Phusion Hot Start II High fidelity DNA polymerase from Thermo Fisher Scientific) on the pEGFP-N2 RNAi plasmid and by primer synthesis on siRNA encoding insert sequences (see Supplementary Table 1). Asymmetrical PCR products, containing double stranded LIC sites either at their 5′ or at their 3′ end, were treated with DpnI to eliminate circular template plasmids and denatured by heating and reannealed to obtain linearized double-stranded pEGFP-N2 RNAi vectors with 5′ single stranded LIC sequences on both strands. Primers corresponding to siRNA sequences, spacers and complement were annealed to obtain inserts with overhanging 5′ LIC sites. Vector and inserts were hybridized and introduced without prior *in vitro* ligation into competent *E.coli* bacteria. Colonies obtained after *in vivo* ligation of the vector and inserts were screened by PCR to verify insert insertion. Recombinant vectors were prepared from positive colonies and sequenced in the siRNA encoding region. PCR and sequencing primer sequences are shown on Supplementary Table 1.

Empty pEGFP-N2 RNAi plasmids and WNK1 or scramble shRNA encoding vectors were introduced into cells by nucleofection with the Amaxa 4D-Nucleofector™ System (Lonza) and Primary Cell Nucleofector™ Solution P3. Transfection efficiency ($EGFP^+$ cells) was monitored using the IncuCyte® (ESSEN BioScience) live cell imaging system after transfection. Preliminary experiments were performed to determine the optimal transfection pulse.

Protein extract preparation and Western blotting

Protein extract preparation from TG1 and TG1-C1 GSC transfected with the pEGFP-N2 RNAi vector or with pEGFP-N2 RNAi WNK1 shRNA or scramble shRNA -expressing plasmids as well as Western blotting conditions were as described in the Materials and Methods section of the main manuscript. Preparation of protein extracts and Western blotting on control (0.1% DMSO alone) proliferating TG1 and TG1-C1 cells or the same cells treated for 6 h with increasing concentrations (0.1, 1, 10 and 100 µM) of the WNK kinase inhibitor WNK463 (purchased from Selleckchem Euromedex) in 0.1% DMSO, were also performed as described in the main manuscript.

Immunocytochemistry and confocal microscopy

Proliferating and quiescent (8 days without medium renewal) TG1 and TG1-C1 GSC were transiently transfected with the pCIneo-3FLAG vectors encoding WT and mutant forms of WNK1 (T60A, T60D, T60E and K233M) as described in the Materials and Methods section in the main manuscript. 24 hours post-transfection, cells were collected, washed 3 times with PBS (phosphate-buffered saline) and deposited on Superfrost microscope slides (CARL Roth) with the Cytospin™ Centrifuge (5 min 600 rpm). Then, cells were fixed in 4% paraformaldehyde (PFA) (Thermo Fisher Scientific) in PBS for 10 min at room temperature (RT). After washing with PBS, cells were permeabilized with

0.2% Triton X-100 in PBS for 10 min at RT, washed with PBS and non-specific sites were saturated during a 1-hour incubation in PBS containing 3% BSA (Bovine Serum Albumin). Slides were then incubated with primary mouse anti-FLAG M2 antibody (1:250) from Sigma-Aldrich in PBS 1% BSA for 2 hours at RT. Washing with PBS was followed by an incubation with anti-mouse Alexa 568 antibody (Molecular Probes) (1:250 in PBS 1% BSA) for 45 min at RT, washing with PBS, DAPI staining of nuclei in PBS (1:500) for 20 min at RT and slide mounting in Fluoromount-G (SouthernBiotech). Images were captured with a Leica SPE confocal microscope equipped with an ORCA-ER chilled CCD camera (Hammamatsu) and the Openlab software (Improvision).

EdU incorporation measurements by flow cytometry

EdU (5-ethynyl-2′-deoxyuridine) incorporation ability of proliferating TG1 and TG1 GSC stably overexpressing WT and mutant forms of WNK1 (Empty vector, WT, T60A, T60D, T60E and K233M) and induced to quiescence was performed with the Click-iT EdU Flow Cytometry Assay Kit from Invitrogen.

REFERENCE

1. Zeniou M, Feve M, Mameri S, Dong J, Salome C, Chen W, El-Habr EA, Bousson F, Sy M, Obszynski J, Boh A, Villa P, Assad Kahn S, et al. Chemical Library Screening and Structure-Function Relationship Studies Identify Bisacodyl as a Potent and Selective Cytotoxic Agent Towards Quiescent Human Glioblastoma Tumor Stem-Like Cells. PLoS One. 2015; 10: e0134793. https://doi.org/10.1371/journal.pone.0134793.

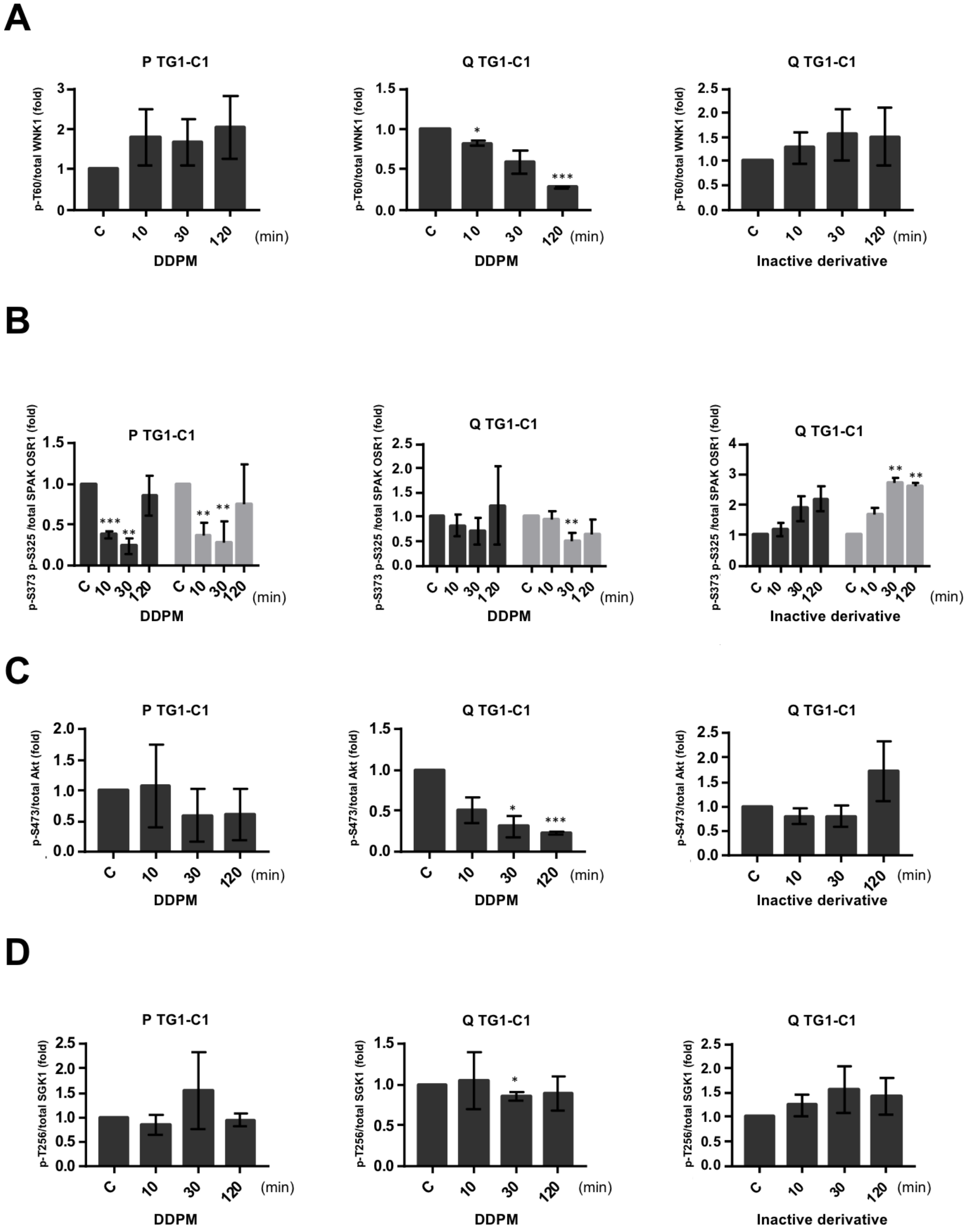

Supplementary Figure 1: DDPM induces modifications of the phosphorylation status of WNK1 and WNK1 partners Akt, SGK1 and SPAK/OSR1 in TG1-C1 GSC. (**A**–**D**) Relative levels of phospho (p)-T60/total WNK1 (A), p-S373 SPAK/p-S325 OSR1/total SPAK (dark grey bars) or p-S373 SPAK/p-S325 OSR1/total OSR1 (light grey bars) (B), p-S473/total Akt (C) and p-T256/total SGK1 (D) were determined using Western blotting and appropriate antibodies in protein extracts from proliferating (P) and quiescent (Q) TG1-C1 GSC treated with DMSO (1%, C: control) or with DDPM (10 µM in 1% DMSO) for 10, 30 or 120 minutes (left and middle panels, respectively). Quiescent (Q) TG1-C1 GSC were also treated with 1% DMSO (C) or with an inactive derivative of DDPM (10 µM in 1% DMSO) for 10, 30 or 120 minutes (right panels). GAPDH expression was used as a loading control. Corresponding quantification plots are shown for each condition (± SEM; $n \geq 2$). Statistical analysis was performed by pairwise comparison of results at each time point relatively to the levels in control (C) conditions used for normalization. Student *t*-test. $^{*}p < 0.05$, $^{**}p < 0.005$, $^{***}p < 0.0005$.

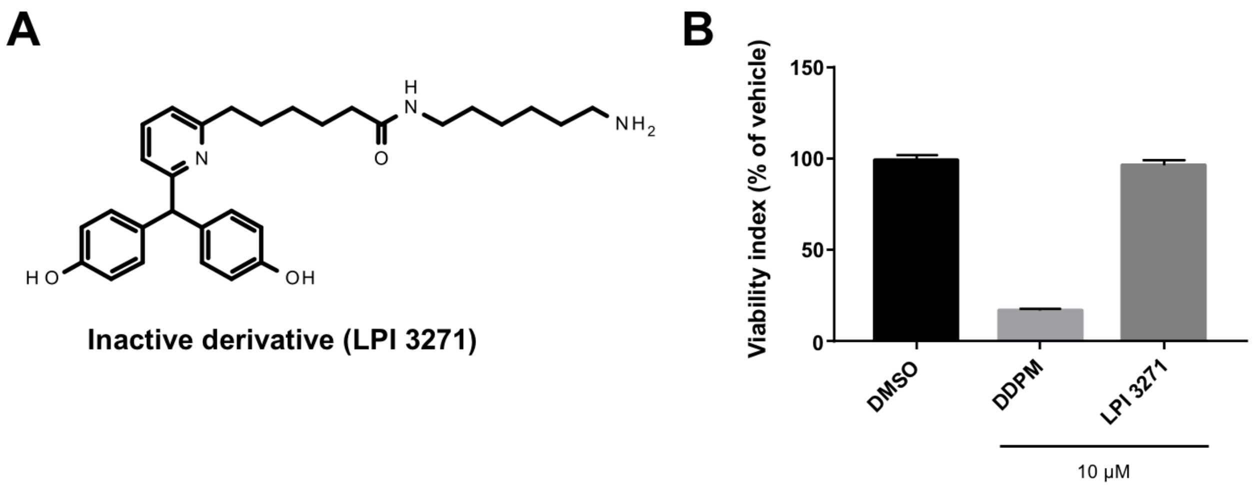

Supplementary Figure 2: Chemical structure and activity profile of DDPM inactive derivative on quiescent GSC. (**A**) Chemical structure of DDPM inactive derivative (LPI3271) used in the study. (**B**) Activity profile of DDPM (10 µM) and DDPM inactive derivative (LPI3271; 10 µM) on quiescent TG1 GSC. Cell viability was evaluated after 24 hours of treatment with the ATP-Glo cell viability assay. Results are mean (± s.d.) in biological triplicates from one experiment out of three independent experiments giving similar results.

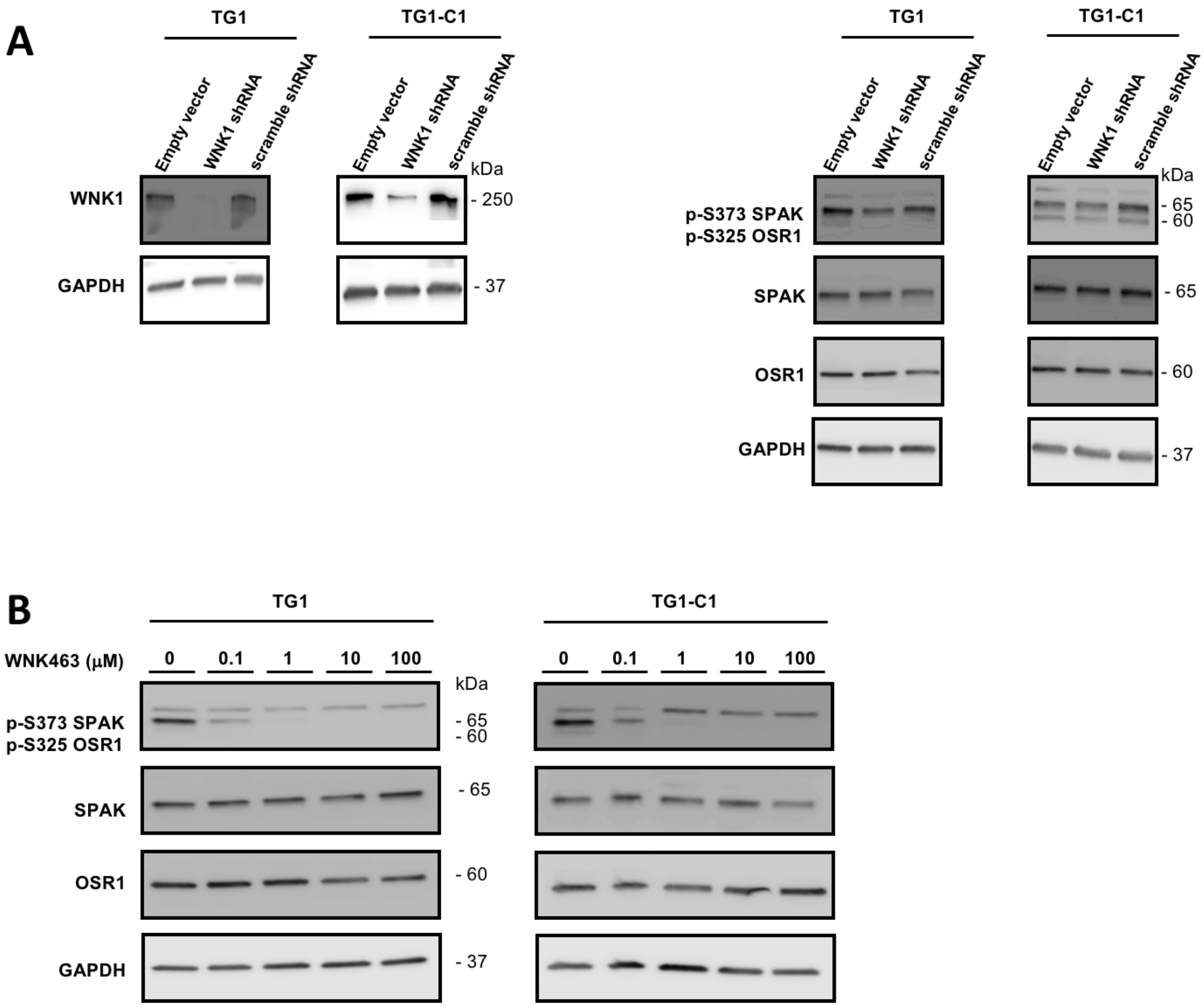

Supplementary Figure 3: SPAK and OSR1 are WNK protein substrates in TG1 and TG1-C1 GSC. (**A**) Proliferating TG1 and TG1-C1 GSC were transiently transfected with the pEGFP N2 RNAi vector (Empty vector) or with the same vector allowing expression of shRNAs directed against WNK1 (WNK1 shRNA) or of non-targeting scramble shRNAs (scramble shRNA). 48 hours post-transfection, WNK1, p-S373 SPAK and p-S325 OSR1 as well as SPAK and OSR1 protein levels were analyzed by Western blotting with GAPDH as loading control ($n = 3$). (**B**) Proliferating TG1 and TG1-C1 GSC were treated for 6 h in the presence of DMSO alone (0,1%) or in the presence of increasing concentrations (0.1, 1, 10 and 100 µM) of the WNK inhibitor WNK463 in 0.1% DMSO. Following protein extraction, SPAK, OSR1 and p-S373 SPAK and p-S325 OSR1 protein levels were determined by Western blotting. GAPDH was again used as a loading control ($n = 2$). Partial reduction of WNK1 expression results in a slight decrease of the phosphorylation status of SPAK and OSR1 proteins on residues S373 (SPAK) and S325 (OSR1). A dose-dependent loss of the phosphorylation of these residues is observed both in TG1 and TG1-C1 cells treated with the WNK kinase activity inhibitor WNK463, suggesting that SPAK and OSR1 proteins are WNK substrates in GSC.

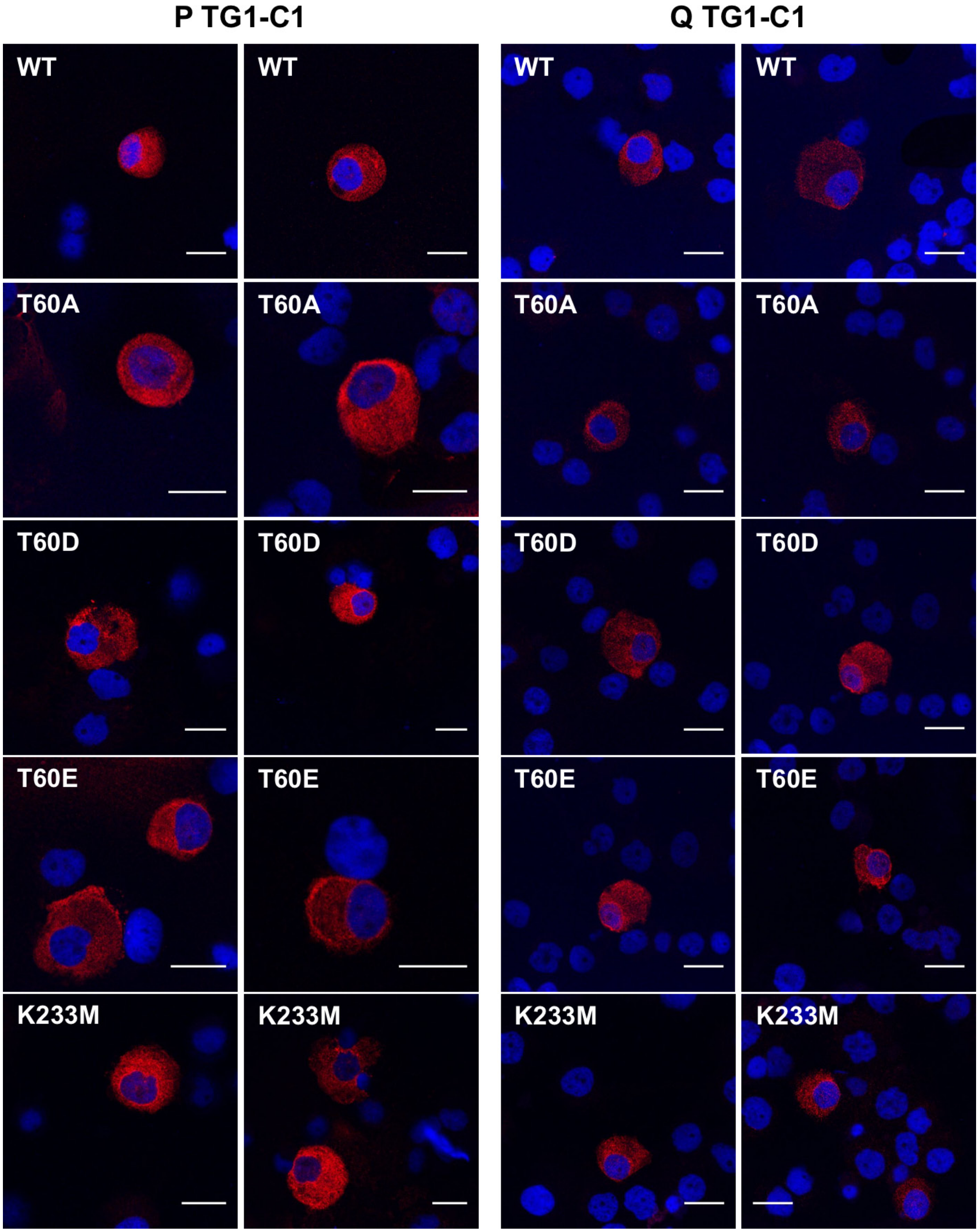

Supplementary Figure 4: WT and mutant FLAG-WNK1 subcellular localization in proliferating and quiescent GSC. Proliferating (P) and quiescent (Q) TG1-C1 GSC were transiently transfected with the pCIneo-3FLAG Gateway vectors allowing overexpression of 3FLAG-tagged WT and mutant (T60A, T60D, T60E, K233) WNK1 isoforms. The presence and subcellular localization of overexpressed proteins (red) was determined by anti-FLAG primary antibody staining detected with an Alexa Fluor 568-congugated secondary antibody and immunofluorescence analysis with a confocal microscope. Nuclei were stained with DAPI. Scale bars: 20 µm.

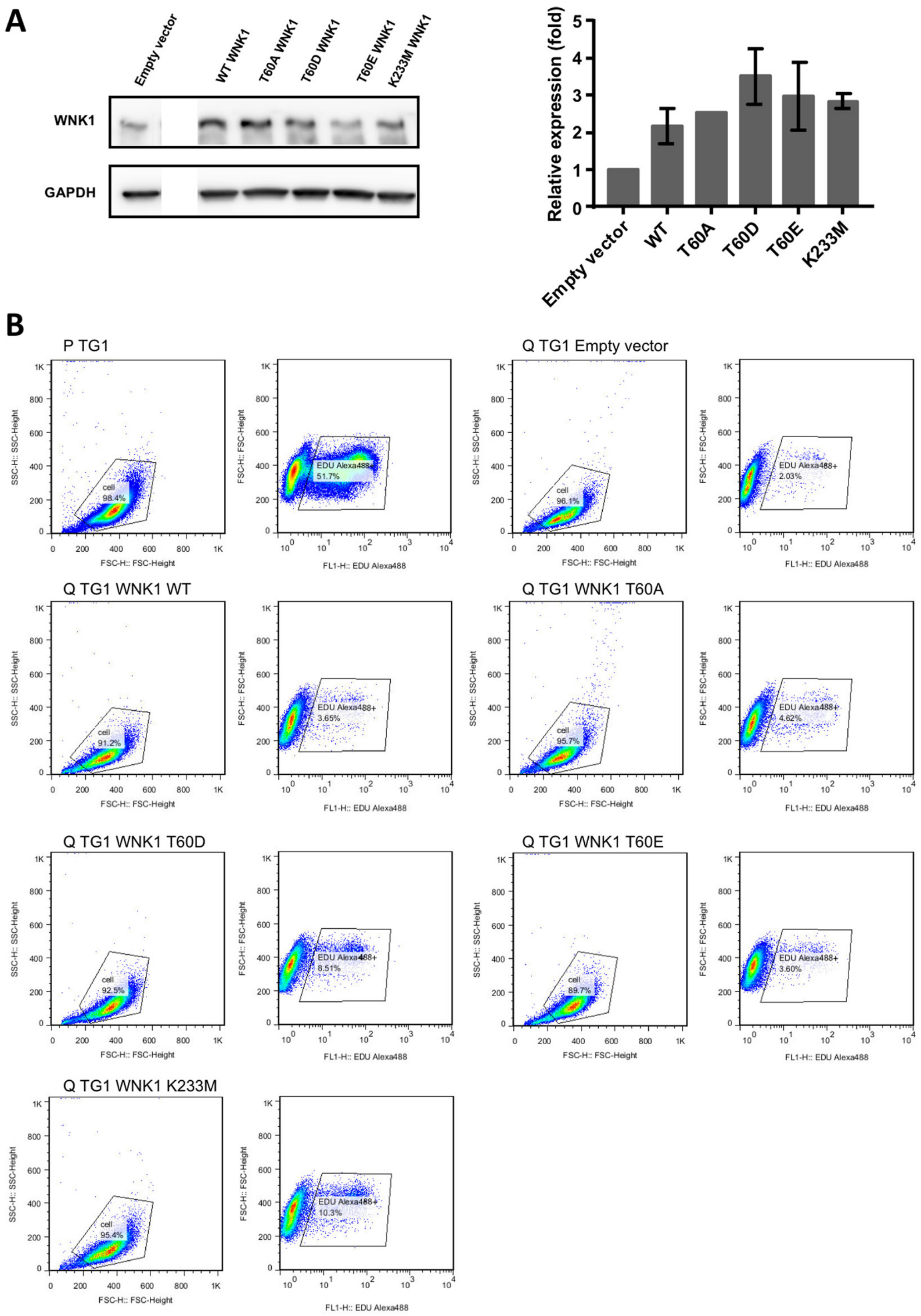

Supplementary Figure 5: Stable cell lines derived from transfected TG1 GSC overexpress WT and mutant WNK1 proteins. Overexpression of WT and mutant WNK1 does not influence EdU incorporation properties of these cells in quiescent culture conditions. (**A**) WNK1 levels were evaluated by Western blotting with an anti-WNK1 antibody in protein extracts from proliferating TG1 GSC stably transfected with pCIneo-3FLAG Gateway vectors in which cDNAs encoding WT and mutated (T60A, T60D, T60E, K233M) WNK1 proteins were cloned. GAPDH was used as a loading control. Empty lane corresponds to an additional sample which was removed from the gel. Quantification of pixel intensity (corrected to GAPDH) and expressed as fold expression relative to control cells stably transfected with the corresponding empty vector, is shown. Results are expressed as mean (± SEM) from two independent experiments. (**B**) EdU incorporation ability was measured by flow cytometry in proliferating (P) TG1 GSC and in TG1 GSC stably transfected with Empty vector or with constructs overexpressing wildtype (WT) and mutant forms of WNK1 (T60A, T60D, T60E, K233) induced to quiescence in the absence of medium renewal for 9 days. The percentage of cells incorporating EdU is indicated on the flow cytometry plot for each condition.

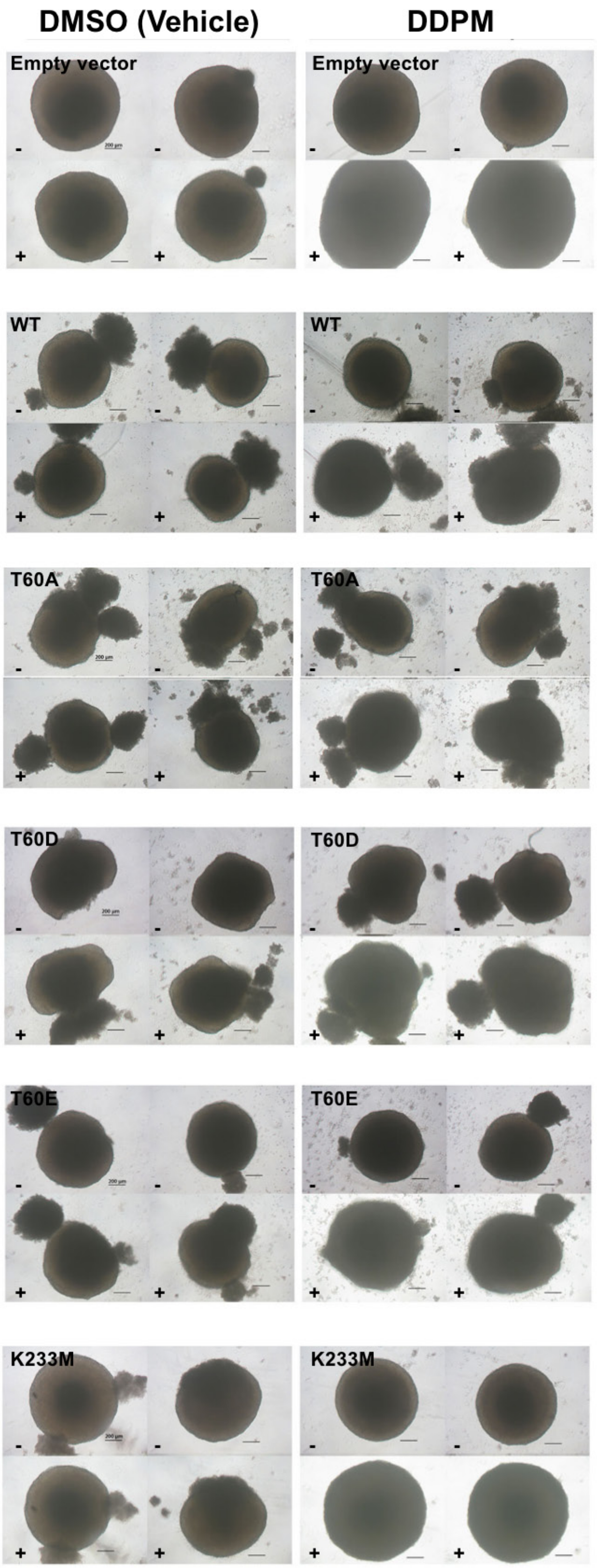

Supplementary Figure 6: Macro-spheres derived from TG1 cells stably transfected with empty vector or constructs allowing overexpression of WT and mutant forms of WNK1 prior to and after treatment with DDPM or vehicle. Macro-spheres derived from control (Empty vector-transfected) TG1 cells or TG1 cells stably overexpressing WT or mutant (T60A, T60D, T60E, K233M) WNK1 proteins were either treated with vehicle (DMSO, left panels) or with DDPM (3 µM in DMSO, right panels) for 24 hours. Two independent spheres are shown for each type of treatment. -: no treatment; +: treatment with DMSO alone or DDPM in DMSO. Scale bars: 200 µm.

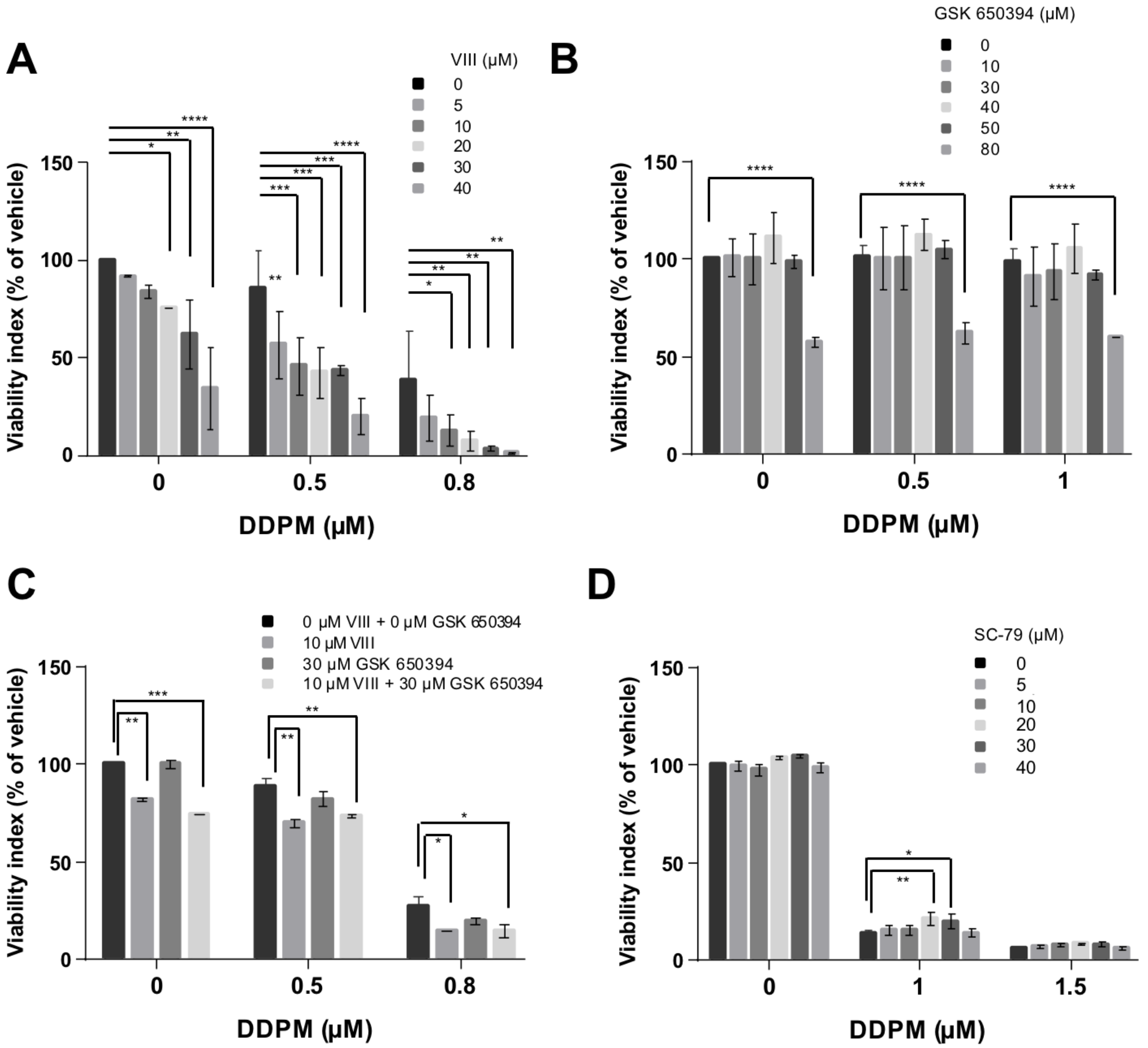

Supplementary Figure 7: Modulation of Akt and SGK1 impacts DDPM cytotoxicity on quiescent TG1-C1 GSC. (**A–C**) Quiescent TG1-C1 GSC were either mock-treated (1% DMSO) or incubated in the presence of DDPM at indicated concentrations and/or Akt inhibitor VIII (A), SGK1 inhibitor GSK 650394 (B) or a combination of both compounds (C) for 24 hours. Cell viability measurements were performed with the ATP-Glo cell survival- based assay. Results are expressed as a percentage of the viability of control TG1-C1 GSC incubated with 1% DMSO alone for 24 hours. Results are the mean of two independent experiments performed in triplicate (± SEM). Statistical analysis was performed with Two-way ANOVA and Tukey's multiple comparisons test. $^{*}p < 0.05$, $^{**}p < 0.005$, $^{***}p < 0.0005$; $^{****}p < 0.0001$. (**D**) Quiescent TG1-C1 GSC were incubated in 1% DMSO (controls) or pretreated for 24 hours with the indicated concentrations of the Akt activator SC-79. Indicated concentrations of DDPM alone (in 1% DMSO) were then added and treatment was pursued for 24 hours. Control cells were maintained in 1% DMSO. Cell viability assays, presentation of results and statistical analysis were performed as described in A, B and C ($n = 3$, mean ± SEM). $^{*}p < 0.05$, $^{**}p < 0.005$.

A

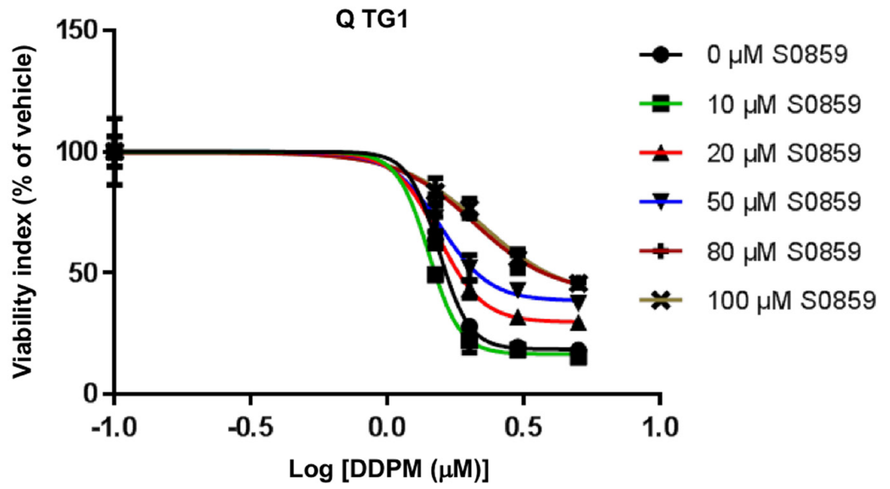

	0 µM S0859	10 µM S0859	20 µM S0859	50 µM S0859	80 µM S0859	100 µM S0859
IC50	1.543	1.415	1.538	1.564	2.073	2.21

µM

B

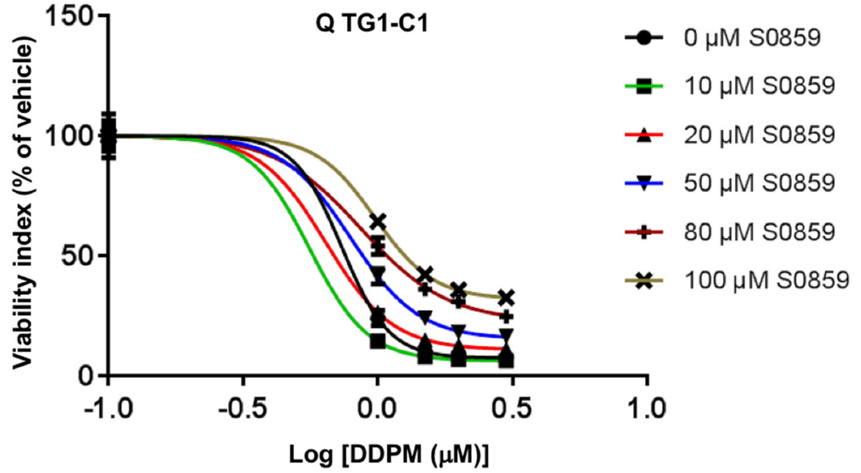

	0 µM S0859	10 µM S0859	20 µM S0859	50 µM S0859	80 µM S0859	100 µM S0859
IC50	0.7353	0.5578	0.6427	0.7916	0.8657	0.977

µM

Supplementary Figure 8: Inhibition of $Na^+/HCO3^-$ NBC cotransporter function partially protects quiescent GSC from DDPM cytotoxic action. (**A**, **B**) Quiescent (Q) TG1 (A) or TG1-C1 (B) GSC were pretreated with DMSO alone (1%) or increasing concentrations (10, 20, 50, 80 and 100 µM) of S0859 (24 hours of treatment). Increasing concentrations of DDPM were subsequently added. Cell viability was evaluated 24 hours later with the ATP-Glo cell viability assay. The fitted sigmoidal logistic dose-response curves to ATP-Glo cell survival assay readouts are shown. Results are mean (± s.d.) in biological triplicates from one experiment out of three independent experiments giving similar results. IC_{50} (concentration resulting in 50% of inhibition) values (µM) are indicated below the graphs.

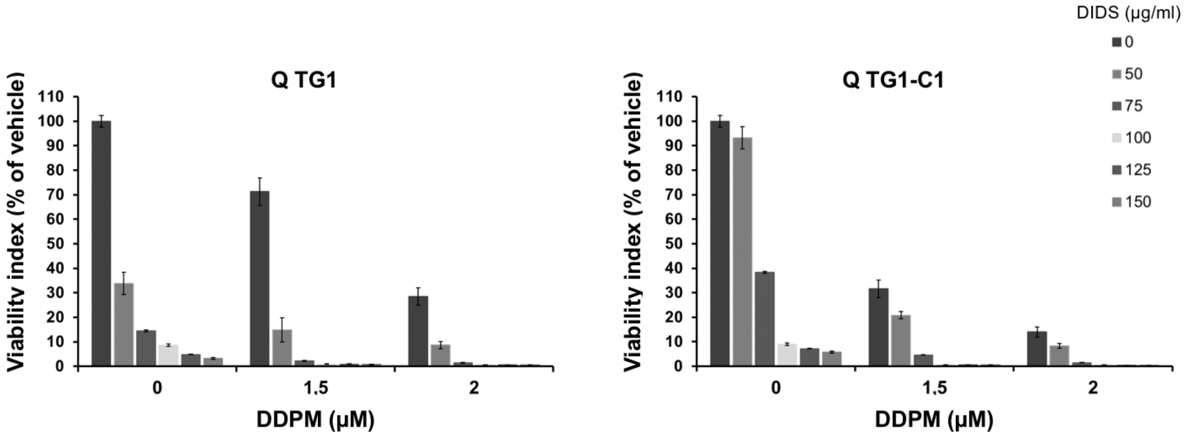

Supplementary Figure 9: The non-selective anion transport inhibitor DIDS is highly cytotoxic to quiescent GSC and does not protect these cells from DDPM cytotoxicity. Quiescent (Q) TG1 (left panel) and TG1-C1 (right panel) GSC were pre-treated for 24 hours with increasing concentrations of the stilbene derivative DIDs inhibiting most Na^+-coupled $HCO3^-$ cotransporters but not NBCn1. Subsequently, cells were mock-treated (1% DMSO and DIDS from the pretreatment) or incubated in the presence of DDPM (1.5 or 2 µM and DIDS from the pretreatment) for 24 hours. Cell viability measurements were performed with the ATP-Glo cell survival-based assay. Results are expressed as a percentage of the viability of control TG1 or TG1-C1 GSC incubated with 1% DMSO (vehicle) alone for 48 hours. Results are the mean of two independent experiments performed in triplicate (± SEM).

Supplementary Table 1: List of primers used in the study

DNA	Usage	Sequences
WNK1	PCR for Gateway cloning	Fwd 5′-GGGGACAAGTTTGTACAAAAAAGCAGGCTTCTCTGGCGGCGCCGCAGAGAAG-3′<br>Rev 5′-GGGGACCACTTTGTACAAGAAAGCTGGGTCCTAAGTGGTCCGCAGGTTGGAGCC-3′
WNK1	PCR for mutagenesis (T60A)	Fwd 5′-CAGGCGCCGCCGCCACGCTATGGACAAGGACAGC-3′<br>Rev 5′-GCTGTCCTTGTCCATAGCGTGGCGGCGGCGCCTG-3′
WNK1	PCR for mutagenesis (T60D)	Fwd 5′-CAGGCGCCGCCGCCACGATATGGACAAGGACAGCC-3′<br>Rev 5′-GGCTGTCCTTGTCCATATCGTGGCGGCGGCGCCTG-3′
WNK1	PCR for mutagenesis (T60E)	Fwd 5′-CAGGCGCCGCCGCCACGAGATGGACAAGGACAGCC-3′<br>Rev 5′-GGCTGTCCTTGTCCATCTCGTGGCGGCGGCGCCTG-3′
WNK1	PCR for mutagenesis (K233M)	Fwd 5′-GAAATCGGCAGAGGCTCCTTTATGACGGTCTACAAAGGTCTGG-3′<br>Rev 5′-CCAGACCTTTGTAGACCGTCATAAAGGAGCCTCTGCCGATTTC-3′
WNK1	sequencing	5′-GGGAGCAAAGAGGAGCCG-3′
WNK1	sequencing	5′-CTTGAGATGGCTACATCTG-3′
WNK1	sequencing	5′-CAGACAGTTTCATATGGTTC-3′
WNK1	sequencing	5′-CAGCTCCTACAACCAGCAG-3′
WNK1	sequencing	5′-GGTGACAACCCCGAGGAG-3′
WNK1	sequencing	5′-GTAACTTCAGGTGGTCTC-3′
WNK1	sequencing	5′-CATTGTCCTGAAGTAGATTC-3′
WNK1	sequencing	5′-GTCAGAAGATGCAAAGTCTG-3′
WNK1	sequencing	5′-CAGGGAGAAGACGACGAC-3′
WNK1	sequencing	5′-ATGGACTACAAAGACGATGACG-3′
pDONR 207	sequencing	pDONOR FP: GATC primer
pDONR 207	sequencing	pDONOR RP: GATC primer
pDONR 207	sequencing	CMV-F: GATC primer
WNK1	PCR for colony screening	Fwd: 5′-GAATTCGAACGCTGACGTCATC-3′<br>Rev: 5′-GAACATGACGGTATCGATAAG-3′
WNK1	Primers for WNK1 shRNA encoding insert assembly	Fwd: 5′-GAGACCACAGATCCCCCAATGAGTCAGATATCGAATTCAAGAGATTCGATATCTGACTCATTGTTTTTA-3′<br>Rev: 5′-GGTATCGATAAGCTTAAAAACAATGAGTCAGATATCGAATCTCTTGAATTCGATATCTGACTCATTGGGG-3′
WNK1	Primers for scramble shRNA encoding insert assembly	Fwd: 5′-GAGACCACAGATCCCCCACTACGAATGACGTATAGATTCAAGAGATCTATACGTCATTCGTAGTTTTTTA-3′<br>Rev: 5′-GGTATCGATAAGCTTAAAAAACTACGAATGACGTATAGATCTCTTGAATCTATACGTCATTCGTAGTGGG-3′
pEGFP-N2 RNAi	PCR for pEGFP-N2 RNAi vector with LIC sites assembly	Fwd: 5′- GTCATGTTCTTTCCTGCGTTATC-3′<br>Rev: 5′- GATCTGTGGTCTCATACAGAACTTATAAGATTCCCAAATCC-3′
pEGFP-N2 RNAi	PCR for pEGFP-N2 RNAi vector with LIC sites assembly	Fwd: 5′-AGCTTATCGATACCGTCATGTTCTTTCCTGCGTTATC-3′<br>Rev: 5′-ATACAGAACTTATAAGATTCCCAAATCC-3′
WNK1	ShRNA vector sequencing	5′-GAATTCGAACGCTGACGTCATC-3′
WNK1	ShRNA vector sequencing	5′-GAACATGACGGTATCGATAAG-3′

Supplementary Table 2: List of antibodies used in the study. See Supplementary_Table_2
