# Supplementary material for: WNK1 kinase and its partners Akt, SGK1 and NBC-family Na+/HCO3− cotransporters are potential therapeutic targets for glioblastoma stem-like cells linked to Bisacodyl signaling
Source: Oncotarget. 2018 Jun 5;9(43):27197–219. doi: 10.18632/oncotarget.25509 (PMC6007472; doi:10.18632/oncotarget.25509)
Supplement: Supplementary file 2 [file oncotarget-09-27197-s002.docx]

**Supplementary Table S2. List of antibodies used in the study**

| Name | Ref. Catalogue | Company | Dilution | Reference |
| --- | --- | --- | --- | --- |
| Rabbit polyclonal anti-Akt | #9272 | Cell signaling Technology | WB 1/1000 | (1) |
| Rabbit polyclonal anti-phospho-Akt (Ser473) | #9271 | Cell signaling Technology | WB 1/500-1/1000 | (1) |
| Rabbit polyclonal anti-NBCe1/SLC4A4 | #11867 | Cell signaling Technology | WB 1/1000 | (1) |
| Rabbit polyclonal anti-NBCn1/SLC4A7 | Orb304589 | Biorbyt | WB 1/1000 | - |
| Rabbit polyclonal anti-OSR1 | #3729 | Cell signaling Technology | WB 1/800-1/1000 | (2) |
| Rabbit polyclonal anti-SPAK | #2281 | Cell signaling Technology | WB 1/800-1/1000 | (2) |
| Rabbit polyclonal anti-phospho-SPAK (Ser373) / phospho-OSR1 (Ser325) | 07-2273 | EMD Millipore | WB 1/1000 | (3) |
| Rabbit polyclonal anti-SGK1 | NBP1-32635 | Novus Biologicals | WB 1/900 | - |
| Rabbit polyclonal anti-phospho-SGK1 (Thr 256) | sc-16744 | Santa Cruz Biotechnology | WB 1/500 | (4) |
| Rabbit polyclonal anti-WNK1 | #4979 | Cell signaling Technology | WB 1/500 | (2) |
| Rabbit monoclonal anti-WNK1 | AB174854 | Abcam | WB 1/1000 | - |
| Rabbit polyclonal anti-phospho-WNK1 (Thr60) | #4946 | Cell signaling Technology | WB 1/500 | (5) |
| Rabbit monoclonal anti-GAPDH (D16H11) XP^®^ | #5174 | Cell signaling Technology | WB 1/5000 | (6) |
| Mouse monoclonal anti-Na^+^/K^+^ ATPase | sc-71638 | Santa Cruz Biotechnology | WB 1/1000 | (7) |
| Goat anti-rabbit IgG, HRP-linked | #7074 | Cell signaling Technology | WB 1/5000 | (6) |
| Horse anti-mouse IgG, HRP-linked | #7076 | Cell signaling Technology | WB 1/10000 | (8) |
| Mouse monoclonal anti-FLAG® M2 | F1804 | Sigma-Aldrich | IF: 1:250 | (9) |
| Goat anti-mouse Alexa Fluor 568 IgG | A11031 | Molecular Probes | IF: 1:250 |  |

1. Prasad V, Lorenz JN, Lasko VM, Nieman ML, Al Moamen NJ, Shull GE. Loss of the AE3 Cl(-)/HCO(-) 3 exchanger in mice affects rate-dependent inotropy and stress-related AKT signaling in heart. Front Physiol. 2013; 4:399.
2. Tu SW, Bugde A, Luby-Phelps K, Cobb MH. WNK1 is required fo r mitosis and abscission. Proc Natl Acad Sci USA. 2011; 108:1385–90.
3. Yang SS, Huang CL, Chen HE, Tung CS, Shih HP, Liu YP. Effects of SPAK knockout on sensorimotor gating, novelty exploration, and brain area-dependent expressions of NKCC1 and KCC2 in a mouse model of schizophrenia. Prog Neuropsychopharmacol Biol Psychiatry. 2015; 61:30–36.
4. Chen W, Chen Y, Xu BE, Juang YC, Stippec S, Zhao Y, Cobb MH. Regulation of a third conserved phosphorylation site in SGK1. J Biol Chem. 2009; 284:3453–60.
5. Nimmanon T, Ziliotto S, Morris S, Flanagan L, Taylor KM. Phosphorylation of zinc channel ZIP7 drives MAPK, PI3K and mTOR growth and proliferation signalling. Metallomics. 2017; 9:471–81.
6. Xiao Y, Jiang Y, Song H, Liang T, Li Y, Yan D, Fu Q, Li Z. RNF7 knockdown inhibits prostate cancer tumorigenesis by inactivation of ERK1/2 pathway. Sci Rep. 2017; 7:43683.
7. Lee SJ, Litan A, Li Z, Graves B, Lindsey S , Barwe SP, Langhans SA. Na,K-ATPase β1-subunit is a target of sonic hedgehog signaling and enhances medulloblastoma tumorigenicity. Mol Cancer. 2015; 14:159.
8. Busnelli M, Manzini S, Hilvo M, Parolini C, Ganzetti GS, Dellera F, Ekroos K, Jänis M, Escalante-Alcalde D, Sirtori CR, Laaksonen R, Chiesa G. Liver-specific deletion of the Plpp3 gene alters plasma lipid composition and worsens atherosclerosis in apoE-/- mice. Sci Rep. 2017; 7:44503.
9. Xue J, Zhou A, Tan C, Wu Y, Lee HT, Li W, Xie K, Huang S. Forkhead Box M1 Is Essential for Nuclear Localization of Glioma-associated Oncogene Homolog 1 in Glioblastoma Multiforme Cells by Promoting Importin-7 Expression. J Biol Chem. 2015; 290:18662–70.
